# Systematic comparison of differential expression networks in MTB mono-, HIV mono- and MTB/HIV co-infections for drug repurposing

**DOI:** 10.1371/journal.pcbi.1010744

**Published:** 2022-12-19

**Authors:** Yao Jiang, Jia-Xuan Zhang, Rong Liu

**Affiliations:** Hubei Key Laboratory of Agricultural Bioinformatics, College of Informatics, Huazhong Agricultural University, Wuhan, P. R. China; Fundação Getúlio Vargas: Fundacao Getulio Vargas, BRAZIL

## Abstract

The synergy between human immunodeficiency virus (HIV) and *Mycobacterium tuberculosis* (MTB) could accelerate the deterioration of immunological functions. Previous studies have explored the pathogenic mechanisms of HIV mono-infection (HMI), MTB mono-infection (MMI) and MTB/HIV co-infection (MHCI), but their similarities and specificities remain to be profoundly investigated. We thus designed a computational framework named IDEN to identify gene pairs related to these states, which were then compared from different perspectives. MMI-related genes showed the highest enrichment level on a greater number of chromosomes. Genes shared by more states tended to be more evolutionarily conserved, posttranslationally modified and topologically important. At the expression level, HMI-specific gene pairs yielded higher correlations, while the overlapping pairs involved in MHCI had significantly lower correlations. The correlation changes of common gene pairs showed that MHCI shared more similarities with MMI. Moreover, MMI- and MHCI-related genes were enriched in more identical pathways and biological processes, further illustrating that MTB may play a dominant role in co-infection. Hub genes specific to each state could promote pathogen infections, while those shared by two states could enhance immune responses. Finally, we improved the network proximity measure for drug repurposing by considering the importance of gene pairs, and approximately ten drug candidates were identified for each disease state.

## Author summary

Although many efforts have been devoted to exploring the pathogeneses of MTB mono-infection (MMI), HIV mono-infection (HMI) and MTB/HIV co-infection (MHCI), the similarities and differences among the host responses to these infections are not yet illustrated. To address this issue, we constructed the integrated differential expression networks (IDENs) of the three disease states by combining differential and non-differential interactions. We first showed that the IDEN framework could provide a more comprehensive picture of host responses than its component methods. Then, we compared the genes in the three IDENs from different viewpoints and found that genes shared by more states showed a stronger preference for certain properties. The correlation analysis and enrichment analysis suggested that MHCI was more similar to MMI rather than HMI, implying that co-infection may be mainly dependent on the pathogenesis of TB. Additionally, we found that hub genes specific to each state may facilitate pathogen infections, whereas hub genes shared by two states may strengthen immune defenses. Finally, we designed gene pair-based network proximity measures to prioritize drug candidates for each disease state, which may provide new insights into the treatment of infections caused by MTB and/or HIV.

## Introduction

Tuberculosis (TB) and acquired immune deficiency syndrome (AIDS) are the two most lethal infectious diseases worldwide caused by *Mycobacterium tuberculosis* (MTB) and human immunodeficiency virus (HIV), respectively [1]. The host could be simultaneously infected by MTB and HIV, which promote each other to accelerate the deterioration of immunological functions [2]. HIV infections are the primary risk factor for developing active TB and greatly increase the risk of reactivation of latent TB patients, while MTB infections could accelerate the progression of HIV infections to AIDS and increase the mortality of HIV-infected patients [3,4]. Although intensive efforts have been devoted to investigating the pathogenic mechanisms of the three disease states, namely HIV mono-infection (HMI), MTB mono-infection (MMI) and MTB/HIV co-infection (MHCI) [2], little attention was paid to the similarities and specific differences in the host responses to these states. The increasing transcriptomic data of relevant patients provide unprecedented opportunities to address this problem from a computational perspective. Additionally, the efficacy of existing anti-MTB and anti-HIV drugs becomes less significant, and the specific drugs are lacking for the treatment of co-infections [5]. Therefore, investigation of host responses to these states may provide clues to the repurposing of existing therapeutic agents.

Blood transcriptional profiles could reflect the pathological responses of the host to pathogens and thus have been widely applied to the investigation of MTB or HIV infections. Berry et al. identified 393 transcripts to discriminate between active TB patients and healthy individuals, and these molecules were associated with the type I interferon signaling pathway [6]. Kaforou et al. discovered transcriptional signatures that were converted into disease risk scores to distinguish TB from other conditions in HIV-infected and -uninfected adults and found that these signatures were effective regardless of HIV infections [7]. Devadas et al. compared differentially expressed genes (DEGs) in HIV-1 and HIV-2 infection states. DEGs in HIV-1 infection were involved in critical cellular pathways such as immune cell activation and proliferation, while relatively few DEGs were found in HIV-2 infection [8]. Chen et al. used a meta-analysis strategy to integrate five blood transcriptome datasets and achieve 293 DEGs in co-infection states, four of which could identify HIV patients with or without TB [9]. Based on four TB/HIV co-infection datasets, Duffy et al. used a multi-model machine learning framework to identify 10 transcripts to distinguish disease states associated with co-infection [10]. Generally, these studies extracted gene signatures from expression profiles to identify TB or HIV-related infection states, and the major limitation is that they only focused on individual genes and neglected the relationship between different genes.

Disease progression may be influenced by the rewiring of molecular networks [11]. Thus, integration of blood transcriptional profiles and molecular interactions has been applied to relevant studies. Sambarey et al. combined RNA sequencing data with protein-protein interactions (PPIs) to develop a sensitive network mining algorithm, which identified a signature comprising 10 genes to distinguish between TB patients and other individuals [12]. In addition, they performed a meta-analysis of multiple TB-related blood transcriptome datasets together with PPI networks to discover 380 common core genes that were highly active in TB disease. Kumar et al. identified 275 differentially expressed host factors that regulated MTB load in human macrophages by siRNA screening, and these molecules were functionally associated through dense interactions [13]. Yoon et al. detected the genetic features of individual samples based on co-expression changes of interacting genes, which can favorably distinguish HIV-1 infection stages and reveal stage-specific signatures [14]. Nevertheless, these studies considered the differential co-expression of gene pairs or the differential expression of genes individually. Combining these two types of information could provide a more comprehensive picture of the host immune response networks. For instance, Sun et al. suggested a differential expression network including differential and non-differential interactions to investigate the dynamics of type 2 diabetes [15]. Sun et al. proposed a consistently differential expression network that merged consistently differential and non-differential interactions across multiple microarray datasets to compare active and latent TB infections [16]. Consequently, the similar methodology could be extended to the comparison of MMI, HMI and MHCI. By investigating the available expression data, however, we found that the existing framework cannot be directly applied to our problem. To facilitate the comparison, we needed to develop a customized algorithm according to the characteristics of relevant data.

Regarding drug repurposing, a variety of network-based algorithms have been developed to prioritize potential drugs for different diseases [17–20]. Especially, the network proximity strategy is receiving increasing attention. Guney et al. first demonstrated that the therapeutic effect of a drug can be inferred from the shortest paths between known drug targets and disease proteins in the human PPI network [21]. Zhou et al. used the same proximity measure to quantify the relationship between drug targets and host proteins associated with four human coronaviruses [22]. Peng et al. developed an improved network proximity metric by considering the weight of target genes and evaluated the proximity between more than 5000 molecular drugs and Alzheimer’s disease related genes [23]. However, existing measures only focused on the individual disease proteins and have yet to consider the relationship between drug targets and disease-related protein interactions. Compared to single proteins, as mentioned above, protein interactions may play a more critical role in disease progression. For example, the synergistic effects of amyloid and tau proteins could make a greater impact on Alzheimer’s disease than any protein alone [24]. Moreover, the physical interaction between the primary receptor CD4 and the chemokine receptor CCR5 is essential for HIV-1 entry into host cells [25]. Thus, developing the interaction-based proximity metric may offer novel insights into drug repurposing.

In this study, we developed a computational framework named the integrated differential expression network (IDEN), which combined blood microarray data and human protein interactions to identify gene pairs associated with three disease states (i.e. MMI, HMI and MHCI). First, we constructed the IDEN for each disease state and evaluated the confidence of derived results. Then, we illustrated the similarities and specificities of genes and gene pairs in the three networks from different aspects. Especially, we explored the role of hub genes in subnetworks composed of gene pairs related to different infection states. Finally, we designed network proximity measures by considering the importance of gene pairs and used the metrics to prioritize reusable drugs against the three types of infections. The present work not only improves our understanding of MTB- and/or HIV-infection but also provides novel clues to the treatment of patients with relevant diseases. The data and codes are freely available on GitHub (https://github.com/hzau-liulab/MTB-HIV).

## Materials and methods

### Dataset

#### Blood microarray data

In this work, the gene expression profile datasets were obtained by searching the Gene Expression Omnibus (GEO) database [26]. We adopted ‘tuberculosis’ and ‘HIV’ as the keywords and selected ‘*Homo sapiens*’ and ‘Expression profiling by array’ as the species and study type, respectively. To reduce the influence of batch effects, we only retained the datasets composed of whole blood samples from the GPL10558 platform, because the data of this platform were sufficient to perform meaningful analyses. As shown in S1 Table, we collected four datasets for each disease state (i.e. MMI, HMI and MHCI). Note that the latent TB patients were not included in these datasets. To ensure the similar numerical range of expression profiles, the relevant datasets were processed by log2 transformation and quantile normalization using the *limma* package (S1 Fig) [27]. The probe IDs were converted into gene symbols based on the annotation file, and probes with missing values were excluded from further analyses. When multiple probes corresponded to the same gene, the average value was calculated to represent the gene expression level.

#### Human PPI network

Human PPIs were extracted from the HIPPIE database (v2.2), which collects a total of 411430 PPIs between 18166 proteins from several well-established PPI databases [28]. Self-interactions and duplicates were removed and only PPIs with confidence scores greater than 0 were reserved. Finally, the human PPI network included 400423 unique PPIs comprising 18141 proteins.

#### Drug–target interaction network

The drug-target interactions were extracted from the DrugBank database (v5.17) and the Therapeutic Target Database (TTD, June 2020) [29,30]. We obtained 10770/2506 interactions between 2079/2115 approved drugs and 2575/721 targets from DrugBank/TTD. We merged the data from two databases and only retained the targets present in the human PPIs. A drug-target network having 10061 interactions between 2065 drugs and 2159 targets was constructed in this work.

#### Functionally important gene sets

We collected 212 HIV-related genes involved in the ‘Human immunodeficiency virus 1 infection’ pathway (hsa05170) and 180 TB-related genes involved in the ‘Tuberculosis’ pathway (hsa05152) from the KEGG database. Furthermore, 2828 inflammatory genes were obtained by searching the keyword ‘inflammatory’ in the gene database of NCBI; 1575 human essential genes were extracted from the study of Hart et al. [31]; and 3659 housekeeping genes that were uniformly expressed in all human tissues were derived from the study of Eisenberg and Levanon [32]. We also downloaded virus-human, bacteria-human and fungi-human PPIs from pathogen-host interaction databases (e.g. PATRIC (November 2016), HPIDB, VirHostNet (January 2019) and PHISTO (September 2019)) [33–36]. We achieved 5695, 3394 and 609 targets for viruses, bacteria and fungi, respectively.

### Differential co-expression analysis (DCA)

**Fig 1:**
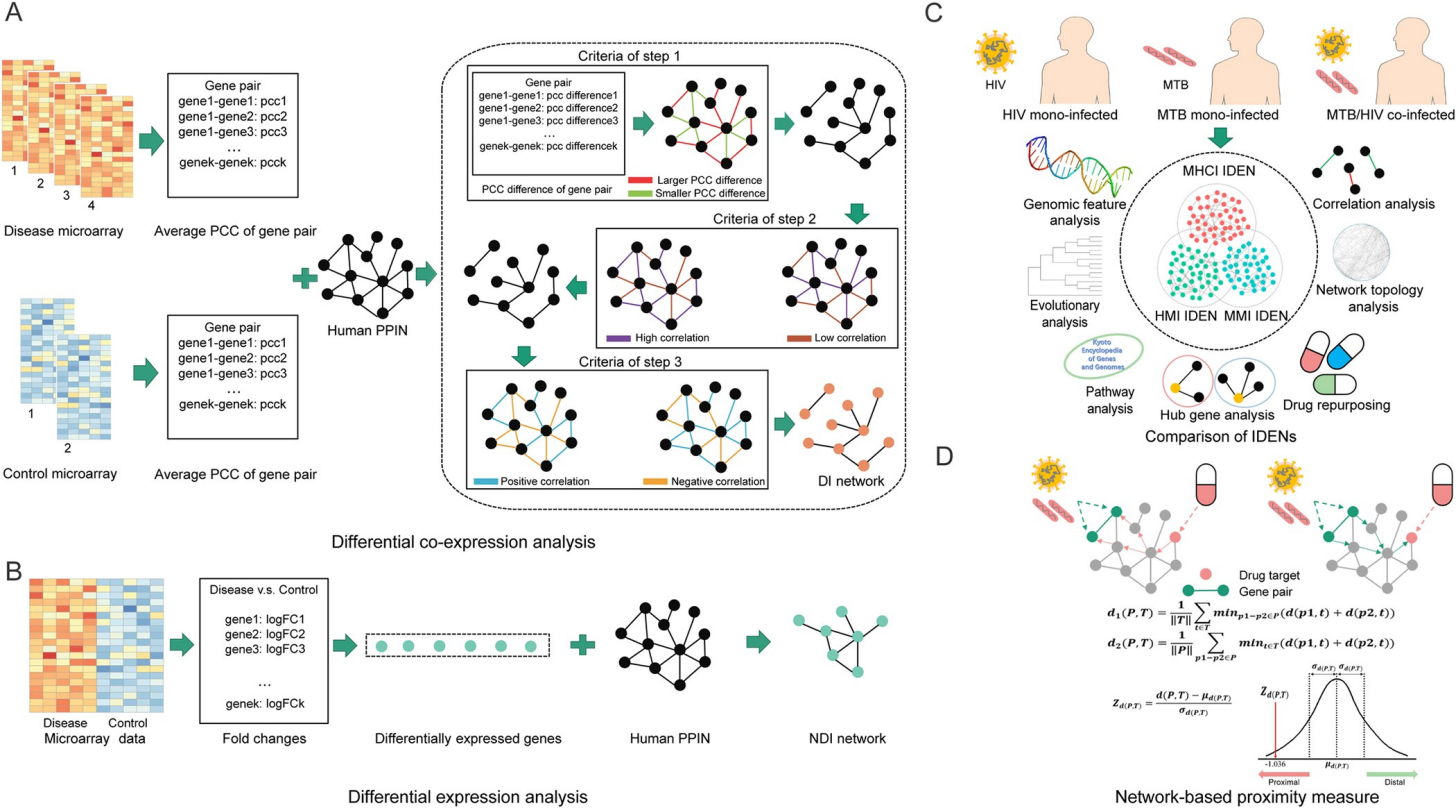
IDEN algorithm and gene pair-based drug repurposing. (A) Flowchart of the DCA method. (B) Flowchart of the DEA method. (C) Comparison of three IDENs. (D) Gene pair-based drug repurposing.

As shown in Fig 1, we evaluated the expression correlation between genes using the Pearson correlation coefficient (PCC). The PCC values of each gene pair in the disease (i.e. MMI, HMI or MHCI) and normal states were calculated. Since the samples associated with each disease state were derived from four datasets (S1 Table), we could obtain four PCC values for each gene pair using the individual datasets. The mean PCC value was calculated as the representative. Similarly, we computed the average PCC value of each gene pair in the healthy group using the GSE29429 and GSE83456 datasets. The resulting PCC values cloud be mapped to the corresponding PPIs, which generated the weighted PPI networks for the disease and normal states, respectively. Then, we computed the difference in PCC values (abbreviated as PCC difference) between the two states for gene pairs and designed a three-step method to extract the gene pairs having significant differential changes. First, we borrowed the idea of the three-sigma rule to identify gene pairs with larger absolute differences. A gene pair was selected if its absolute difference (i.e. |PCC difference|) was greater than the mean plus three standard deviations of |PCC difference| of all gene pairs. Second, we evaluated whether the gene pairs exhibited a difference in the correlation status (high or low correlation). A gene pair was selected if its absolute value of PCC (i.e. |PCC|) was greater than 0.5 (i.e. an empirically assigned cutoff) in one state and less than 0.5 in the other state. Third, we examined whether the gene pairs showed a difference in the sign of correlation values (positive or negative correlation). A gene pair was selected if its PCC was greater than 0 in one state and less than 0 in the other state. The process of three steps is shown in S2 Fig, where the finally reserved gene pairs were defined as differential interactions (DIs). As a control, we randomly permuted the expression values of genes and repeated this procedure 100 times. The one-sample *t*-test was used to evaluate the significance of DIs by comparing the native |PCC difference| with the mean of |PCC difference| derived from random permutations. The results suggested that the *p*-values of all DIs were significant (*p* < 0.05).

### Differential expression analysis (DEA)

In addition to DIs, we identified non-differential interactions (NDIs) involved in various disease states. The NDI generally had trivial changes in expression correlation, but this interaction may also be associated with disease procession if its two genes were DEGs. Because GSE37250, GSE39939 and GSE39940 contained only disease samples and had a clearly different numerical range relative to the normal samples of GSE29429 and GSE83456, these three datasets were excluded from further analyses. In other words, only one dataset could be used for each disease state. For GSE29429 and GSE83456, the DEA procedure was performed directly because they included normal samples. However, there were no normal samples in GSE69581. Considering that GSE69581 and GSE83456 shared a similar numerical range, we combined the samples from these two datasets and removed the batch effect using the ComBat program [37]. Then, the normal samples of GSE83456 could be considered as the control data of GSE69581 (S3 Fig). Based on each dataset, we computed the absolute value of the log2 fold change (i.e. |log_2_FC|) for each gene. Due to the different distribution of |log_2_FC| for the three datasets, a unified threshold may not be suitable for detecting DEGs (S4 Fig), so we continued to adopt the mean plus three standard deviations of |log_2_FC| as the adaptive threshold. Genes with |log_2_FC| greater than this cutoff and FDR < 0.05 were treated as DEGs. Subsequently, DEGs were mapped to the PPI network and a gene pair was defined as a NDI if both genes were DEGs.

### Genomic characteristics analysis

We checked the chromosomal distribution of disease-related genes by calculating the ratio of the number of identified genes on each chromosome to the total number of identified genes. Furthermore, we extracted single nucleotide polymorphisms (SNPs) located in human protein-coding genes from the Ensembl database (release 104) [38]. The SNP density of each gene was defined as the ratio of the number of mapped SNPs to the gene length. Disease-related SNPs (dSNPs) were obtained from the ClinVar database (November 2019) [39]. We computed the proportion of dSNPs among total SNPs in the detected genes. Based on gene-disease associations from the DisGeNET database (v7.0), we assessed the proportion of disease-causing genes among our identified genes [40]. The distances between genes on the same chromosome were also calculated in this work.

### Evolutionary and expression analysis

Here, we investigated three evolutionary characteristics of disease-related genes, including the dN/dS ratio, protein age and homologous gene number. Specifically, human-mouse orthologous genes were derived from the Ensembl BioMart database, and the dN/dS ratio was calculated to illustrate the gene evolutionary rate [38]. The protein age was extracted from the ProteinHistorian database to infer the evolutionary origin of proteins [41]. The homologous gene number was computed based on the HomoloGene database (build68) [42]. Known sites of post-translational modifications (PTMs) in human proteins were derived from the PhosphoSitePlus database (v6.6) [43]. The proportion of modification sites in each protein was calculated to evaluate the PTM level of identified genes.

### Network topology analysis

Based on the human PPI network, we investigated the topological features of identified genes, including the degree centrality, closeness centrality, betweenness centrality and shortest path distribution. The degree centrality is the number of genes that are directly connected to the target gene. The closeness centrality is defined as the inverse of the average shortest path distance from the target gene to other genes in the network. The betweenness centrality is defined as the sum of the fraction of shortest paths between all pairs of genes that pass through the target gene. The shortest path between two genes could be calculated using the Dijkstra algorithm. Furthermore, we adopted the Markov clustering (MCL) algorithm with default parameters to extract topological modules in the PPI network [44]. To evaluate the topological distribution of identified gene pairs, we computed the intramodule to intermodule interaction ratio (MIR = N_intramodule_/N_intermodule_), where N_intramodule_ denotes the number of interactions between two genes from the same module, and N_intermodule_ denotes the number of interactions between two genes from different modules.

### Network proximity measure for drug repurposing

Existing drug repurposing algorithms generally used network-based measures to prioritize potential drugs according to the proximity between known drug targets and disease-related genes in the human PPI network [21]. Considering the importance of gene pairs found in this work, we proposed an alternative strategy to evaluate the effectiveness of drugs by calculating the shortest distance between drug targets (*T*) and disease-related gene pairs (*P*). Here, three interaction-based proximity measures could be designed for each drug as follows:

$$d_{1}(P,T)=\frac{1}{\left\|T\right\|}\sum_{t\in T}\min_{p1-p2\in P}\left(d(p1,t)+d(p2,t\right))$$

$$d_{2}(P,T)=\frac{1}{\left\|P\right\|}\sum_{p1-p2\in P}\min_{t\in T}\left(d(p1,t\right)+d(p2,t))$$

$$d_{3}(P,T)=d_{1}(P,T)+d_{2}(P,T)$$

where *d*(*p*1, *t*) and *d*(*p*2, *t*) denote the shortest distance from genes *p*1 and *p*2 to drug target *t* in the network. The first distance measure is to fix the drug target set and compute the shortest distance to the elements in the PPI set, while the second measure is to fix the PPI set and compute the shortest distance to the elements in the target set. Meanwhile, we constructed a reference distribution for each drug. Referring to the known drug targets, we randomly selected the same number of proteins with similar degree distributions from the whole PPI network. Due to the scale-free nature, a small portion of proteins have extremely high degrees. To avoid the repetitive selection of these proteins, a binning approach was proposed to divide proteins into different intervals such that at least 100 proteins were included in each bin. We repeated the permutation process 1000 times and the original measures were converted into Z-scores as follows:

$$Z_{d(P,T)}=\frac{d(P,T)-\mu_{d(P,T)}}{\sigma_{d(P,T)}}$$

where *μ*_*d*(*P*,*T*)_ and *σ*_*d*(*P*,*T*)_ represent the mean and standard deviation of the results from 1000 permutation tests, respectively. According to the normal distribution, a drug was considered to be potentially effective for a given disease if Z ≤ -1.036 (i.e. a Z-score that was lower than 85% of the reference scores was considered significant in the one-sided test) [45,46].

### Connectivity Map (CMAP) based drug repurposing

Besides the network-based approach, the GSEA program in CMAP was adopted to evaluate the potential of drug candidates by comparing the gene expression profiles upon drug treatment with the gene expression profiles of patients [47]. Because the maximum allowed number of submitted genes is 150, we ranked DEGs based on the |log_2_FC| measure for each disease state and used the top 150 up- and down-regulated DEGs as the gene signature. If the GSEA score is negative (i.e. a negative correlation between the drug and signatures), the drug could reverse the disease. Thus, the GSEA score can be used as an auxiliary metric for assessing the selected drugs. Note that not all drugs can retrieve a score from CMAP.

### Statistical analysis

Hypergeometric tests were adopted to estimate the significance of the overlap of gene sets. For a given attribute, the differences between the two groups of genes or gene pairs were analyzed using Wilcoxon rank sum tests. In this work, *p* < 0.05 was considered statistically significant. All statistical analyses were performed using the R package.

## Results

### Overview of three differential expression networks

**Fig 2:**
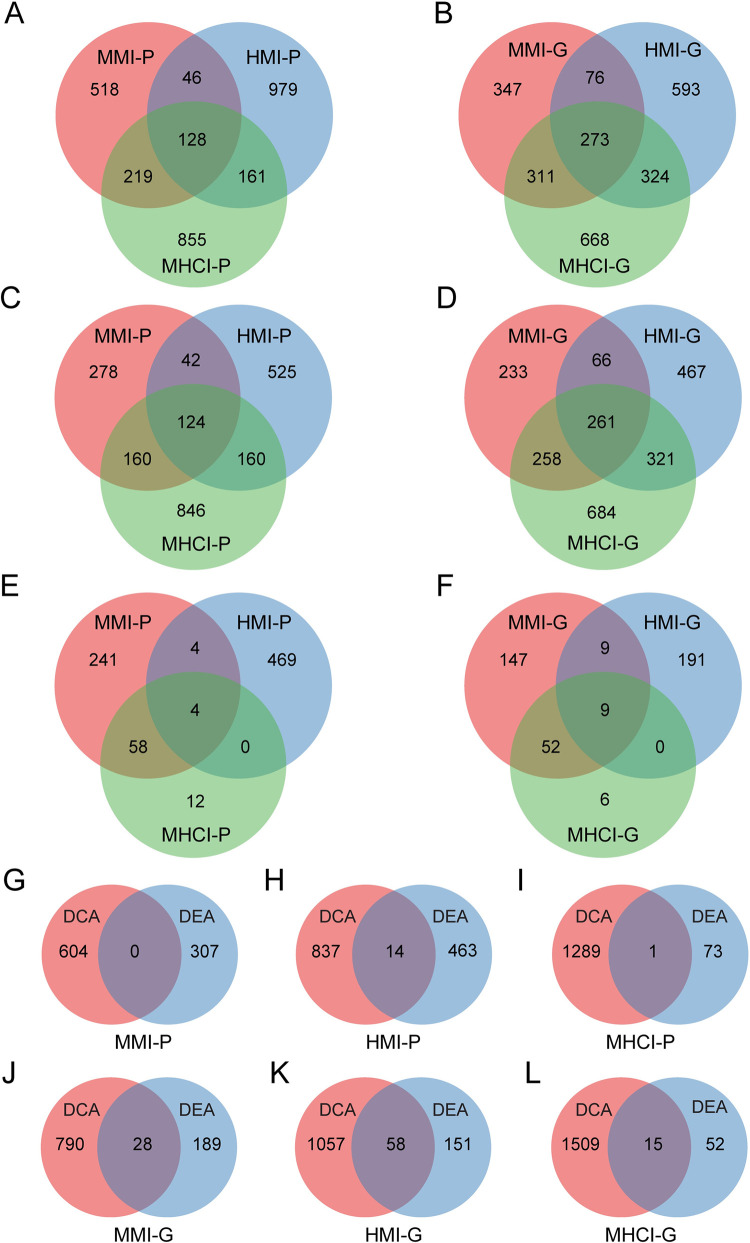
Overview of MMI-, HMI- and MHCI-related gene pairs and genes. (A-B) Number of gene pairs and genes identified by the IDEN framework. (C-D) Number of gene pairs and genes identified by the DCA method. (E-F) Number of gene pairs and genes identified by the DEA method. (G-I) Comparison of gene pairs identified by the DCA and DEA methods. (J-L) Comparison of genes identified by the DCA and DEA methods.

As shown in Fig 1A and 1B, we integrated microarray data and protein interactions to identify gene pairs involved in different pathogen infections. By taking the union of the results of DCA and DEA methods, we obtained the IDEN of MMI comprising 911 gene pairs and 1007 genes (termed MMI-P and MMI-G, respectively), the IDEN of HMI comprising 1314 gene pairs and 1266 genes (termed HMI-P and HMI-G, respectively) and the IDEN of MHCI comprising 1363 gene pairs and 1576 genes (termed MHCI-P and MHCI-G, respectively). We found that there was a significant overlap between any two IDENs (Fig 2A and 2B), suggesting that the three disease states could be related and share common pathogenic mechanisms. Particularly, the overlaps between the MHCI-related and MMI-/HMI-related IDENs were more remarkable than those between the MMI-related and HMI-related IDENs. This indicated that the co-infected state could be more closely associated with two mono-infected states. The DCA method found 604, 851 and 1290 DIs for MMI, HMI and MHCI, respectively, which were the major components of IDENs (Fig 2C). The identical DIs between the two mono-infected states were clearly less than those between the mono-infected and co-infected states, which can explain the above observation for the whole IDENs. The DEA method identified 307, 477 and 74 NDIs for three states (Fig 2E). The identical NDIs between MMI and MHCI accounted for 83.8% (62/74) of the total NDIs of MHCI, while those between HMI and MHCI only accounted for 5.4% (4/74). This result indicated that MHCI would be more similar to MMI from the viewpoint of gene expression changes, implying that TB disease plays a dominant role in co-infection. We also compared the results inferred by the DCA and DEA methods and found that there were small overlaps (Fig 2G–2L). Thus, the IDEN that was a combination of DIs and NDIs may provide more comprehensive insights into host immune responses.

To facilitate the comparison and analysis, the gained genes and gene pairs were separated into different categories. All the genes and gene pairs associated with the three disease states were termed IDEN-G and IDEN-P, respectively (S5 Fig). We divided IDEN-G into seven gene groups, including HMI-specific genes (HMI-SG), MMI-specific genes (MMI-SG), MHCI-specific genes (MHCI-SG), shared genes between HMI and MMI (HMI-MMI-SG), shared genes between HMI and MHCI (HMI-MHCI-SG), shared genes between MMI and MHCI (MMI-MHCI-SG), and common genes among the three disease states (Common-G). The similar partition was performed for IDEN-P. After excluding IDEN-G and IDEN-P from the human PPI network, the remaining genes and gene pairs were considered the control genes and gene pairs, namely Control-G and Control-P.

### Advantage and confidence of the IDEN framework

**Fig 3:**
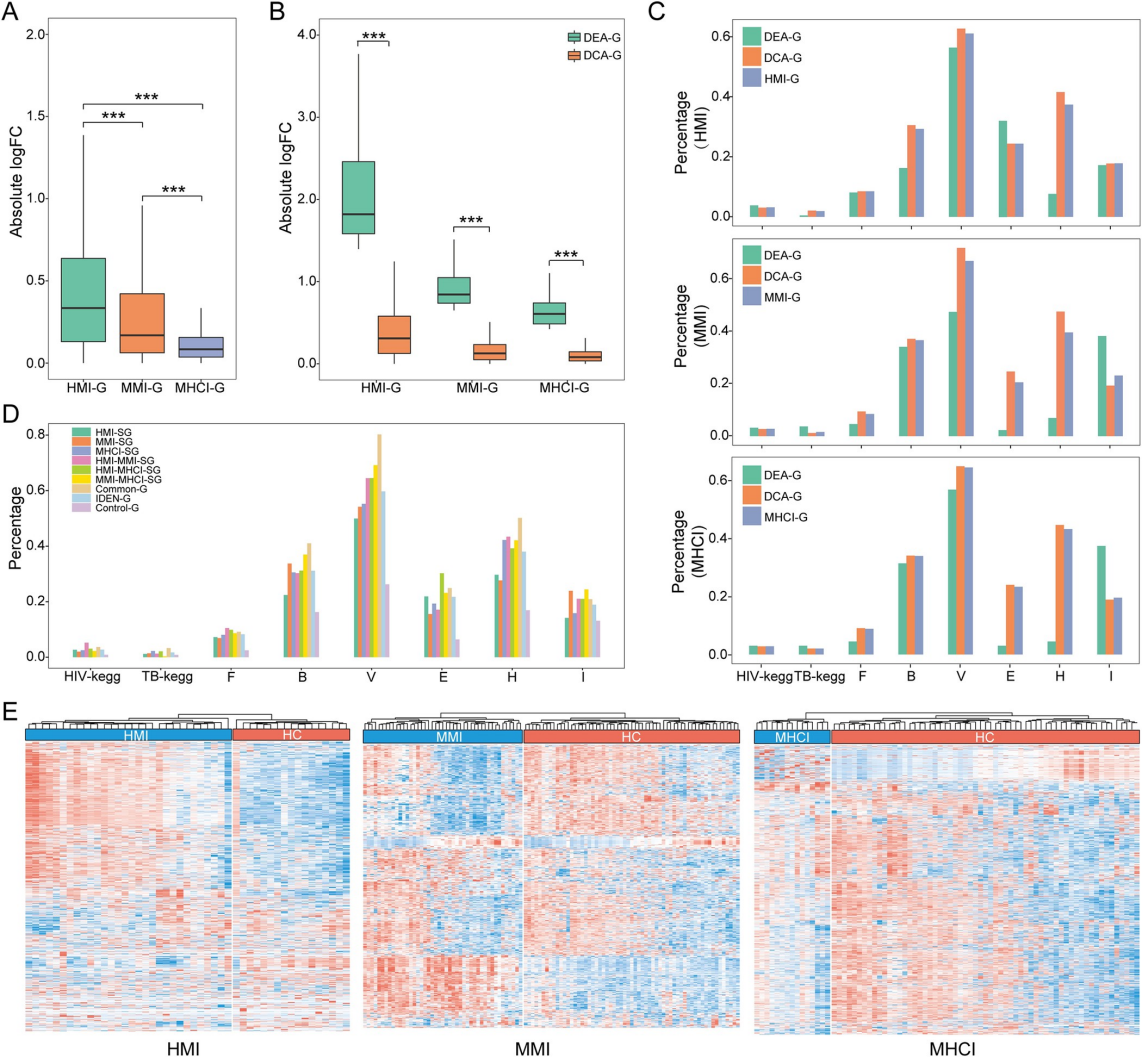
Advantage and confidence of IDEN framework. (A) Expression change of identified genes. (B) Expression change of genes identified by the DCA and DEA methods. (C) Enrichment of genes identified by the DCA and DEA methods in the important gene sets. F, B and V denote human targets for fungi, bacteria and viruses, respectively, and E, H and I denote essential genes, housekeeping genes and inflammatory genes, respectively. (D) Enrichment of the subclasses of IDEN-G in the important gene sets. (E) Clustering analysis of disease and healthy samples. The rows denote disease-related genes (red: up-regulated and blue: down-regulated) and the columns denote samples. *** P<0.001, ** 0.001≤ P<0.01 and * 0.01≤ P<0.05.

To evaluate the biological relevance of the identified genes, we collected genes associated with HIV or MTB infections from existing computational and experimental resources. There were significant overlaps between the three group of genes (i.e. MMI-G, HMI-G and MHCI-G) and the available resources (S2 Table), indicating that the inferred genes were highly involved in the relevant diseases. Then, we compared the expression changes of these genes between disease and normal states (Fig 3A and 3B). HMI-G showed the greatest changes, followed by MMI-G, and MHCI-G showed the smallest changes. During the co-infected state, the expression levels of disease-related genes had trivial changes, but their expression correlations may change dramatically. For each status, genes identified by the DEA (termed DEA-G) had greater changes, whereas those identified by the DCA (termed DCA-G) had relatively mild changes (|log_2_FC|<0.5). Thus, the DCA (which depends on expression correlation changes) could complement the shortcoming of the DEA (which focuses on DEGs).

Moreover, we profoundly compared the results derived from the two component methods. S3 Table displays the enrichment of DCA-G and DEA-G in the seven gene sets including different functional genes, namely HIV- and TB-related genes in KEGG pathways, human target genes of various pathogens (e.g. fungi, bacteria and viruses) and other important host genes (e.g. essential genes, housekeeping genes and inflammatory genes). Compared with DEA-G, DCA-G was generally more enriched in these gene sets (Fig 3C), indicating that the DCA has advantages in identifying potential disease genes associated with pathogen infections. Furthermore, the IDEN identified more valuable genes than the DCA in all gene sets (S3 Table), suggesting that the DEA can also complement the DCA and was indispensable for the proposed framework. Additionally, we explored the relationship between these gene sets and the different subclasses of IDEN-G (S4 Table). IDEN-G and its subclasses showed consistently higher enrichments than Control-G (Fig 3D). Common-G yielded the highest measure for four gene sets, including TB pathway genes, human target genes of bacteria and viruses and housekeeping genes, indicating that Common-G may play a key role in the three disease states.

To further evaluate the confidence of our results, the identified genes and gene pairs were adopted as signatures to distinguish between disease and control samples. Specifically, the expression values of MMI-G, HMI-G and MHCI-G were utilized as features to construct the random forest models using the Scikit-learn package. All parameters except the number of trees (n_estimators = 500) were default values. We evaluated our classifiers using the leave-one-out cross-validation. All the three groups of genes showed favorable performances, and MHCI-G achieved the greatest area under the curve (AUC = 0.993, S5 Table). In addition, we checked the prediction capabilities of MMI-P, HMI-P and MHCI-P by implementing Zhang et al.’s algorithm [48]. Generally, the performances of gene pair-based models were comparable to the results of gene-based models, and MHCI-P yielded the best performance (AUC = 1, S5 Table). We also compared the IDEN signatures with seven gene signatures in previous studies. Our signatures performed more favorably on the identification of MHCI than other signatures and achieved slightly better or comparable performance on the prediction of MMI and HMI (S6 Table). We then performed the clustering analysis based on the gene signatures. Compared with the healthy samples, the disease samples displayed clearly different expression patterns (Fig 3E), again demonstrating the reliability of our results.

### Genomic characteristics analysis

To characterize the achieved genes at the genomic level, we first explored their chromosomal distributions. The analysis illustrated that these disease-related genes were not restricted within specific chromosomes but widely distributed in the human genome. Approximately 97% of these genes were located on autosomes (S6A Fig). Moreover, MMI-G, HMI-G and MHCI-G demonstrated the highest enrichment on 8, 5 and 10 chromosomes, respectively (S1 Data). SNPs are the most prevalent genetic variants in the human genome. We computed the SNP density of genes in the three groups. The SNP density of MMI-G was higher than the measure of the other two gene groups on 9 chromosomes, and MHCI-G and HMI-G displayed the highest density on 7 and 7 chromosomes, respectively (S6B Fig). The distribution of disease SNPs showed that MMI-G, HMI-G and MHCI-G achieved the highest proportion on 11, 7 and 5 chromosomes, respectively (S6C Fig). Also, we investigated the involvement of three gene sets in human diseases using known disease-causing genes as the reference. The proportion of disease-causing genes among our genes on each chromosome was more than 50%. MMI-G obtained a higher proportion on 11 chromosomes than the other two groups (S6D Fig). We also checked the relative positions of gained genes in the genome. The distances of genes from the seven subsets of IDEN-G were significantly different (S7 Fig). In particular, MMI-SG had the closest genomic distance, implying that they tended to be clustered together on the same chromosome.

### Evolutionary and expression analysis

**Fig 4:**
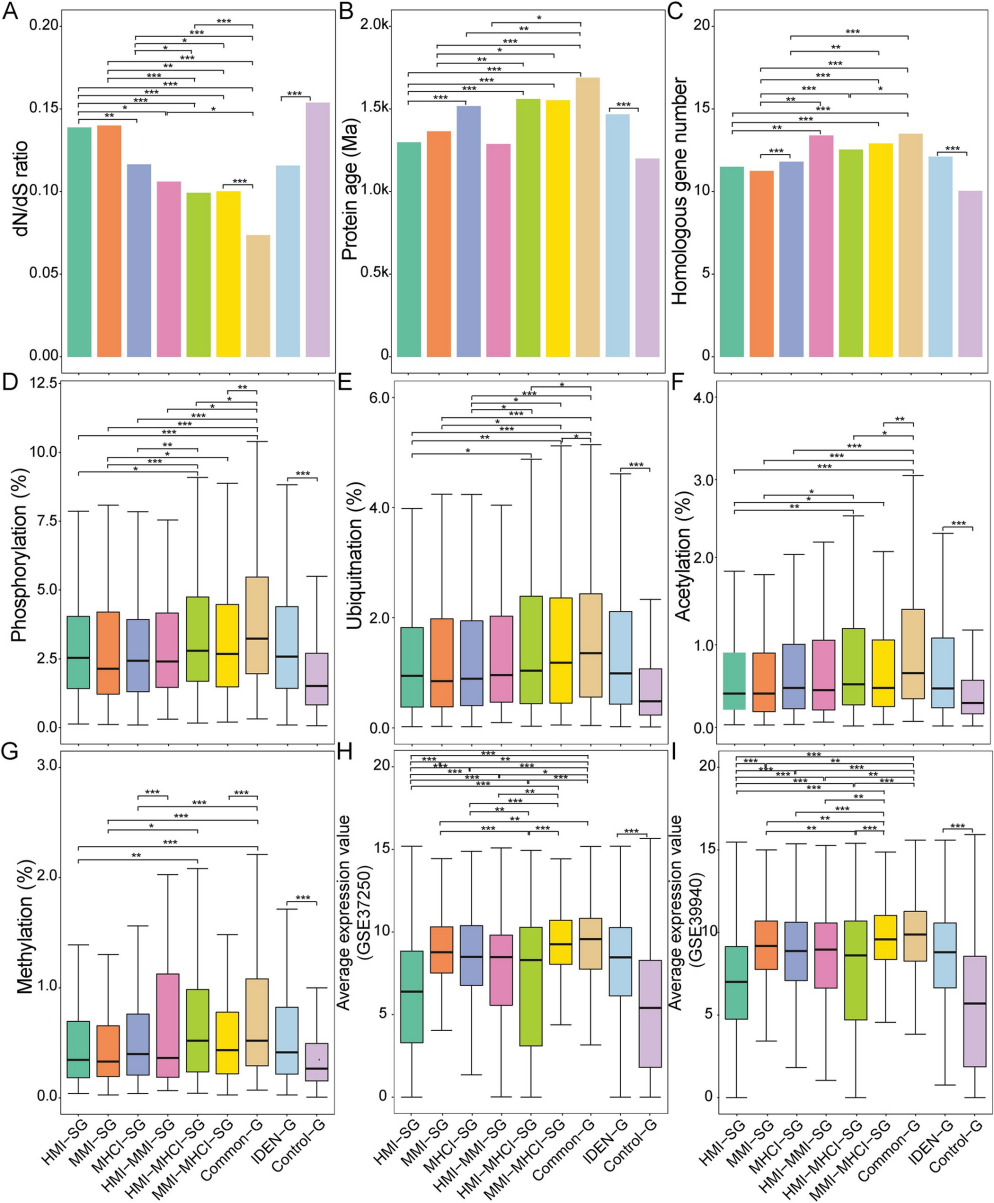
Evolutionary and expression features of disease-related genes. (A) dN/dS ratio. (B) Protein age. (C) Number of homologous genes. (D) Proportion of phosphorylated sites. (E) Proportion of ubiquitinated sites. (F) Proportion of acetylated sites. (G) Proportion of methylated sites. (H) Expression level of detected genes (GSE37250). (I) Expression level of detected genes (GSE39940). *** P<0.001, ** 0.001≤ P<0.01 and * 0.01≤ P<0.05.

In this section, we investigated the evolutionary properties of genes detected by the IDEN framework. IDEN-G possessed lower dN/dS ratios than Control-G and thus showed a stronger purifying selection ability (Fig 4A). Moreover, IDEN-G held older protein ages and higher homologous gene numbers (Fig 4B and 4C). These results were consistent with previous findings that pathogens tended to target relatively conserved genes in the host [49]. Among the seven subclasses, Common-G showed the strongest evolutionary measures, implying that common genes may play indispensable roles in the three infection statuses. And genes shared by two statuses (i.e. HMI-MMI-SG, HMI-MHCI-SG and MMI-MHCI-SG) showed more conserved measures than genes specific to each status (i.e. HMI-SG, MMI-SG and MHCI-SG). Thus, genes with a greater overlapping degree could be more functionally important from an evolutionary viewpoint. Regarding the PTM levels, the protein products of IDEN-G had a higher proportion of phosphorylated, ubiquitinated, acetylated and methylated residues (Fig 4D–4G), implying that disease-related genes probably disrupt cellular activities through different PTMs. Actually, modifications of the host proteome were often manipulated by pathogens to influence the outcome of infections [50]. Additionally, we observed that genes shared by more statuses had more PTM sites.

Based on the two datasets from different references (GSE37250 and GSE39940), the average expression value of all samples in each dataset was computed to represent the expression level of each gene. IDEN-G was more abundantly expressed compared to Control-G (Fig 4H and 4I). Additionally, Common-G held the highest expression level among all subclasses, followed by MMI-MHCI-SG, while HMI-SG had the lowest level. The differences among the other subclasses were not so significant. We divided all samples into different groups (i.e. HMI, MMI and MHCI) and observed similar expression patterns (S8 Fig), implying that the mechanisms of gene expression may be shared by the three states.

### Network topology analysis

**Fig 5:**
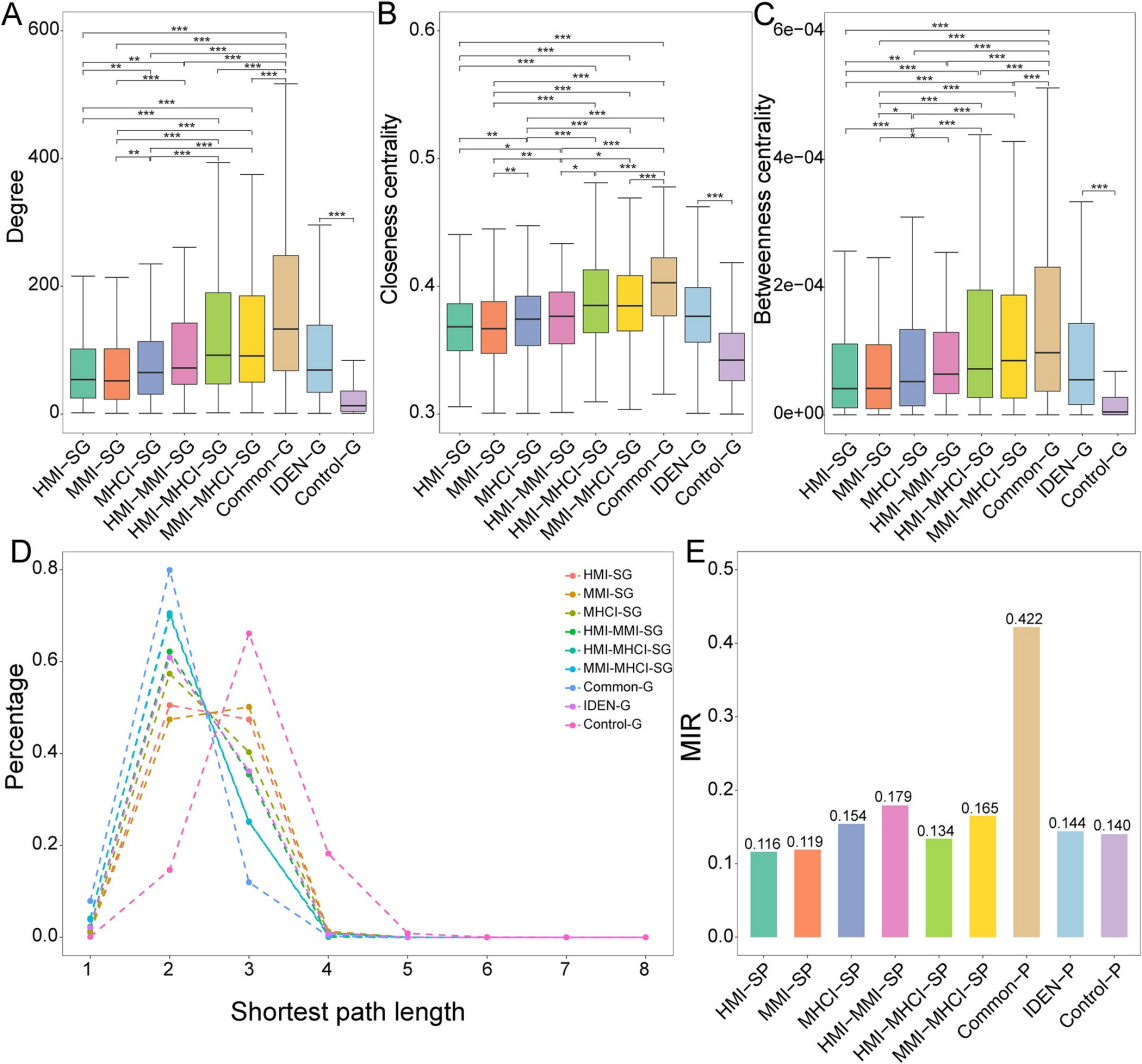
Network topology features of disease-related genes. (A) Degree centrality. (B) Closeness centrality. (C) Betweenness centrality. (D) Shortest path length. (E) MIR measure. *** P<0.001, ** 0.001≤ P<0.01 and * 0.01≤ P<0.05.

To understand the network context of disease-related genes, we computed three topological features, including degree centrality, closeness centrality and betweenness centrality. The degree of IDEN-G was significantly greater than that of Control-G (Fig 5A), suggesting that pathogens may manipulate relevant pathways by targeting highly connected genes. Compared with Control-G, IDEN-G had higher closeness measures, indicating that these genes could be close to the center of the PPI network and easily transmit signals to other relevant genes (Fig 5B). The greater betweenness measures suggested that IDEN-G was located on the shortest paths and can therefore control the information flow among genes (Fig 5C). We also found that genes shared by more statuses had an increasing value for these topological features, illustrating that these genes occupied more important positions in the network.

We then computed the distribution of shortest paths between genes. Compared to Control-G, IDEN-G generally had shorter shortest paths, suggesting that these genes tended to co-localize and form modules in the PPI network (Fig 5D). Especially, this tendency was more remarkable for the genes with a greater overlapping degree (e.g. Common-G and HMI-MHCI-SG). Besides, the intramodule to intermodule interaction ratio was computed to evaluate the topological distribution of gene pairs. We obtained 601 modules consisting of 12518 genes by the MCL algorithm [44]. The MIR of common pairs (Common-P) reached 0.422 (Fig 5E), indicating that the relevant genes were tightly clustered within topological modules, possibly due to their critical roles in the pathogenesis. MHCI-SP, HMI-MMI-SP and MMI-MHCI-SP also achieved relatively higher MIR values than Control-P. Accordingly, the overlapping genes and gene pairs tended to be more adjacent in the network.

### Expression correlation analysis

**Fig 6:**
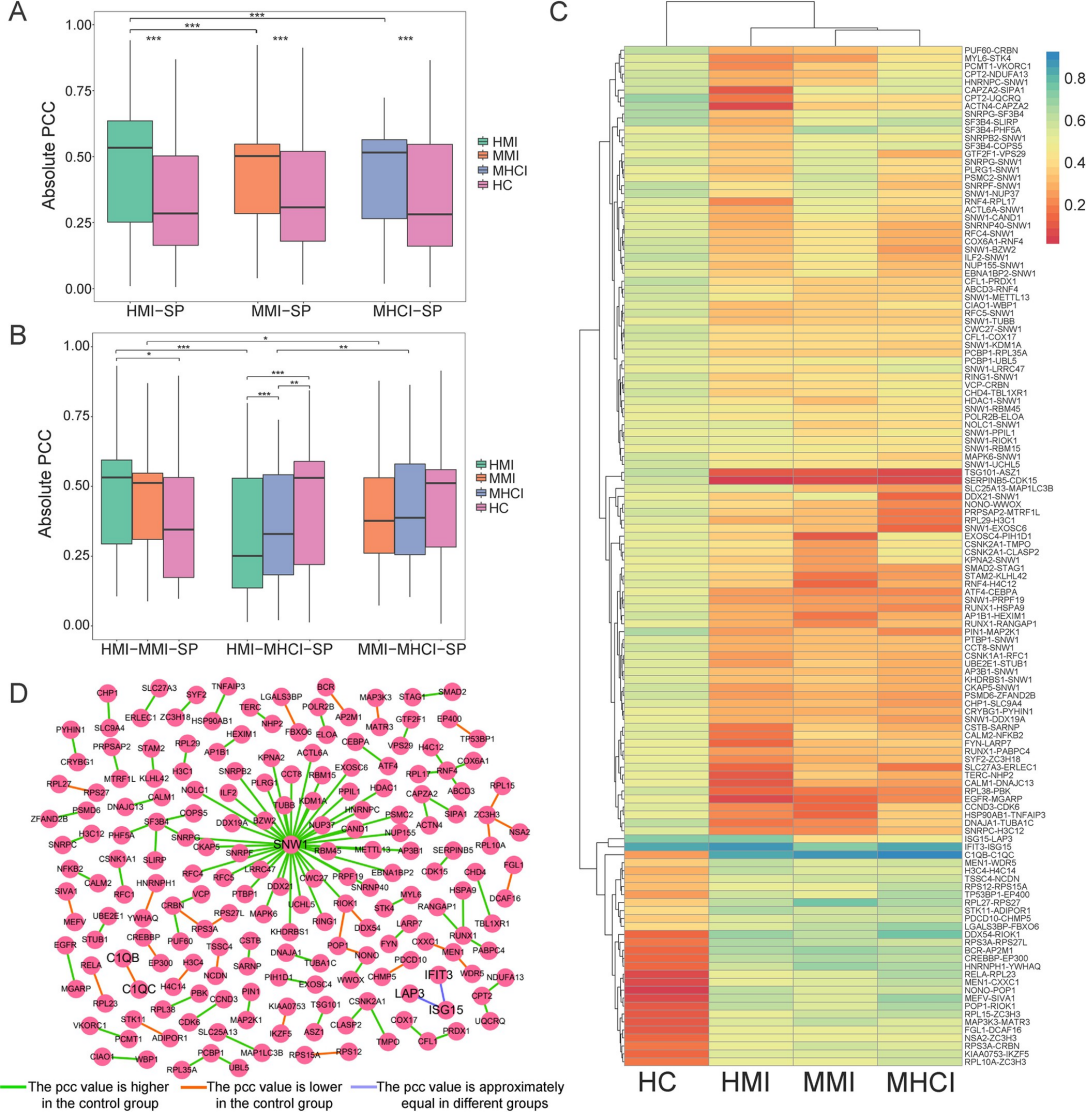
Expression correlations of disease-related gene pairs. (A) Absolute PCC values of specific gene pairs. (B) Absolute PCC values of gene pairs shared by two statuses. (C) Absolute PCC values of common gene pairs. (D) Network of common gene pairs. If the absolute PCC values are consistently greater than (or less than) 0.5 in different groups, the expression correlation of a gene pair is considered to be approximately equal. *** P<0.001, ** 0.001≤ P<0.01 and * 0.01≤ P<0.05.

This section focuses on the comparison of gene pairs from the expression level. As shown in Fig 6A, all the three groups of specific gene pairs (i.e. HMI-SP, MMI-SP, and MHCI-SP) obtained higher |PCC| values in the disease group than in the healthy group, indicating that the associated genes were more strongly interconnected during the specific infection stage. The |PCC| value of HMI-SP was significantly higher than that of the other specific groups. This implied that the protein interactions specific to HMI were more activated. Moreover, we found that HMI-MMI-SP had higher |PCC| values in the two disease states (Fig 6B). However, the opposite trend was observed for HMI-MHCI-SP and MMI-MHCI-SP, suggesting that the correlations of shared pairs related to co-infections decreased significantly in the disease states.

Based on the expression correlations of common pairs, we found that MMI and MHCI shared more similarities (Fig 6C). Among Common-P, 27 gene pairs showed high correlations in the disease state and low correlations in the normal state, while 99 gene pairs had an opposite trend. The *C1QA*, *C1QB* and *C1QC* collectively constitute the complement *C1Q*, which is essential to the activation of the classical complement pathway. The correlation of *C1QB-C1QC* was higher and gradually increased in the three disease states (MHCI > MMI > HMI), illustrating that the interactions between complement components were enhanced for MTB-infected individuals, especially the co-infected patients. Interestingly, 40 pairs with lower correlations in the disease state included a multifunctional protein *SNW1* (Fig 6D), which acts by protein interactions, mRNA splicing regulation and transcriptional control [51]. Accordingly, this protein may be the primary host factor targeted by pathogens and the associated interactions would weaken or even disappear in the disease state.

### Comparative analysis of functional pathways

**Fig 7:**
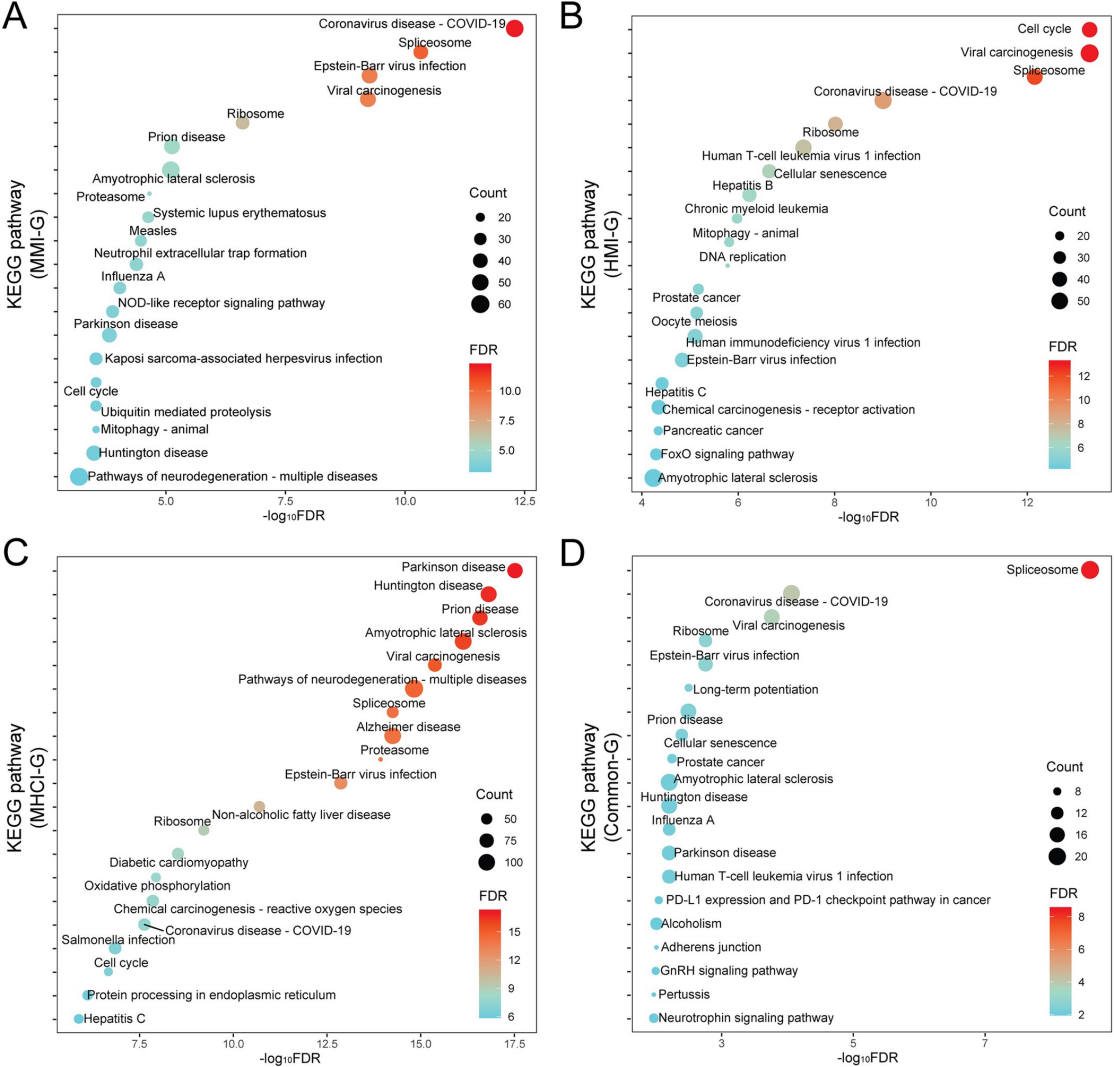
KEGG pathway analysis of disease-related genes. (A) MMI-G. (B) HMI-G. (C) MHCI-G. (D) Common-G.

The KEGG and GO enrichment analysis was performed using the clusterProfiler package [52]. We retained the top 20 pathways with FDR < 0.05. As shown in Fig 7, the three disease states shared seven pathways, such as ‘ribosome’, ‘spliceosome’ and ‘coronvirus disease-COVID-19’. The use of ribosomal pathway was possibly because the host needs to synthesize a large number of proteins in response to pathogen infections [16]. The spliceosome mediates pathogen infections by participating in mRNA splicing, which enables pathogens to prevent the downstream immune responses [53]. Besides, MMI and MHCI shared several additional pathways, such as ‘proteasome’ and ‘prion disease’. The proteasome regulates the degradation of most cellular proteins and removes erroneous proteins [54]. MTB may thus cause immune dysregulation by disturbing the function of proteasomes. In contrast, HMI shared only one pathway with MMI or MHCI. The GO analysis confirmed that the inferred genes were enriched in RNA-related biological processes, and MMI shared more processes with MHCI than HMI (S9 Fig). Overall, MMI was more similar to MHCI according to functional analyses, implying that the pathogenesis of co-infection may be dominated by MTB [55].

**Fig 8:**
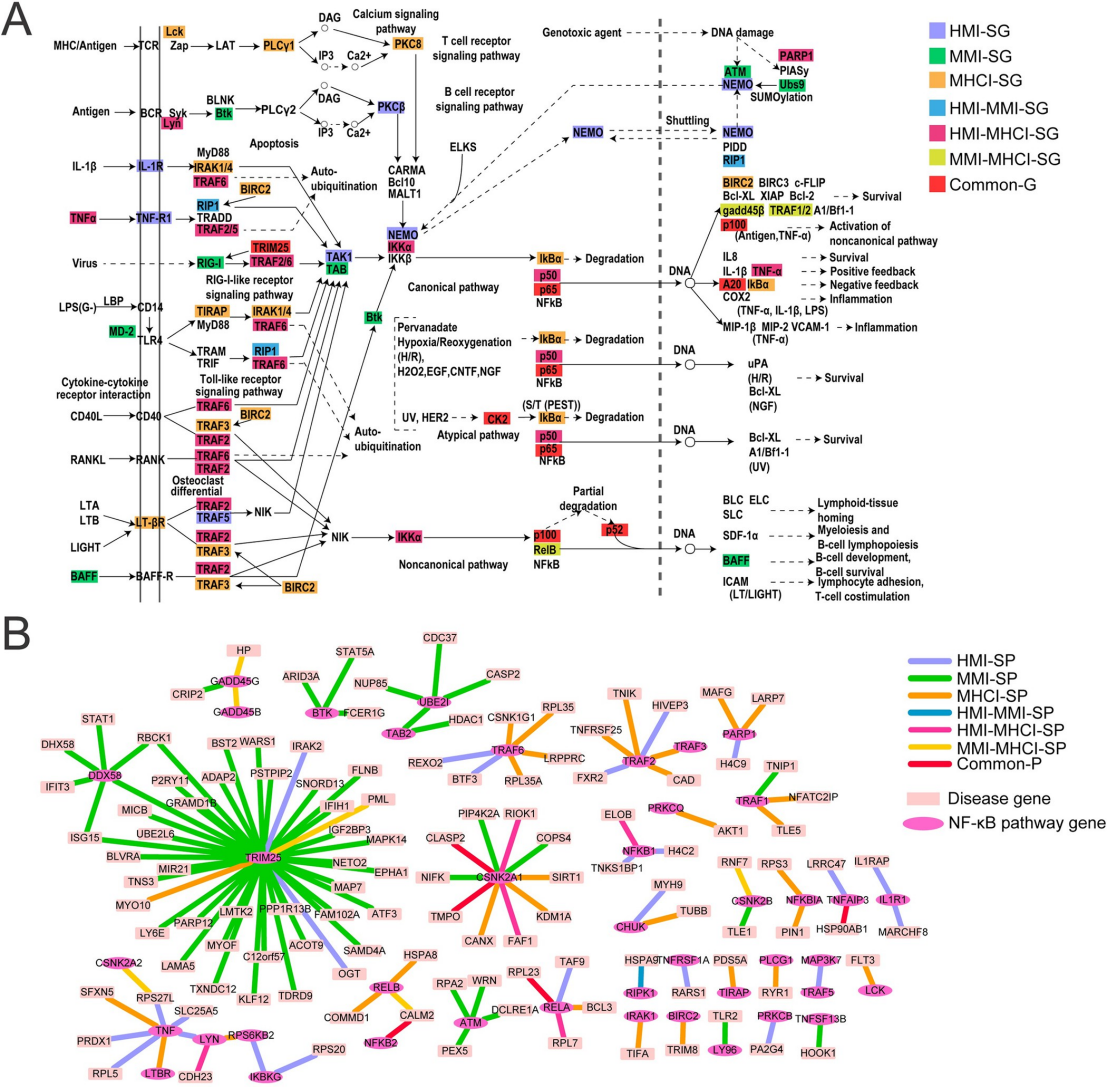
Disease-related genes and gene pairs in NF-κB signaling pathway. (A) Disease-related genes. (B) Disease-related gene pairs.

Nuclear factor-kappa B (NF-κB) was indispensable for the progression of MTB infection and significantly associated with the three disease states (MMI: FDR = 0.003, HMI: FDR = 0.006 and MHCI: FDR = 0.0002), so we profoundly explored this pathway [56]. As shown in Fig 8A, our framework identified 41 genes. Compared to MMI-SG and HMI-SG, MHCI-SG was more widely distributed throughout the pathway and may control the degradation of the suppressor protein IκB. Furthermore, genes in the *TRAF* family (*TRAF2*, *TRAF5* and *TRAF6*) were included in both HMI-SG and HMI-MHCI-SG, implying that these genes may be involved in HIV infection. In fact, the three proteins could interact with HIV-1 Nef proteins to initiate NF-κB activation and increase HIV-1 replication [57]. The p50 and RelB proteins are involved in the formation of NF-κB dimers in the classical and non-classical pathways, respectively. The former was shared by HMI and MHCI, and the latter was shared by MMI and MHCI. This result implied that the p65-p50 dimer may be formed for HIV-infected patients, while the p52-RelB dimer may be generated for MTB-infected patients. Common-G was generally located in downstream positions of the pathway and included p65 and p100, which are also essential for NF-κB dimerization. In Fig 8B, we visualized PPIs related to this pathway. A greater number of MMI-specific pairs (MMI-SP) were observed, further confirming the importance of this pathway in MTB infection.

### Analysis of hub genes in different sub-networks

**Fig 9:**
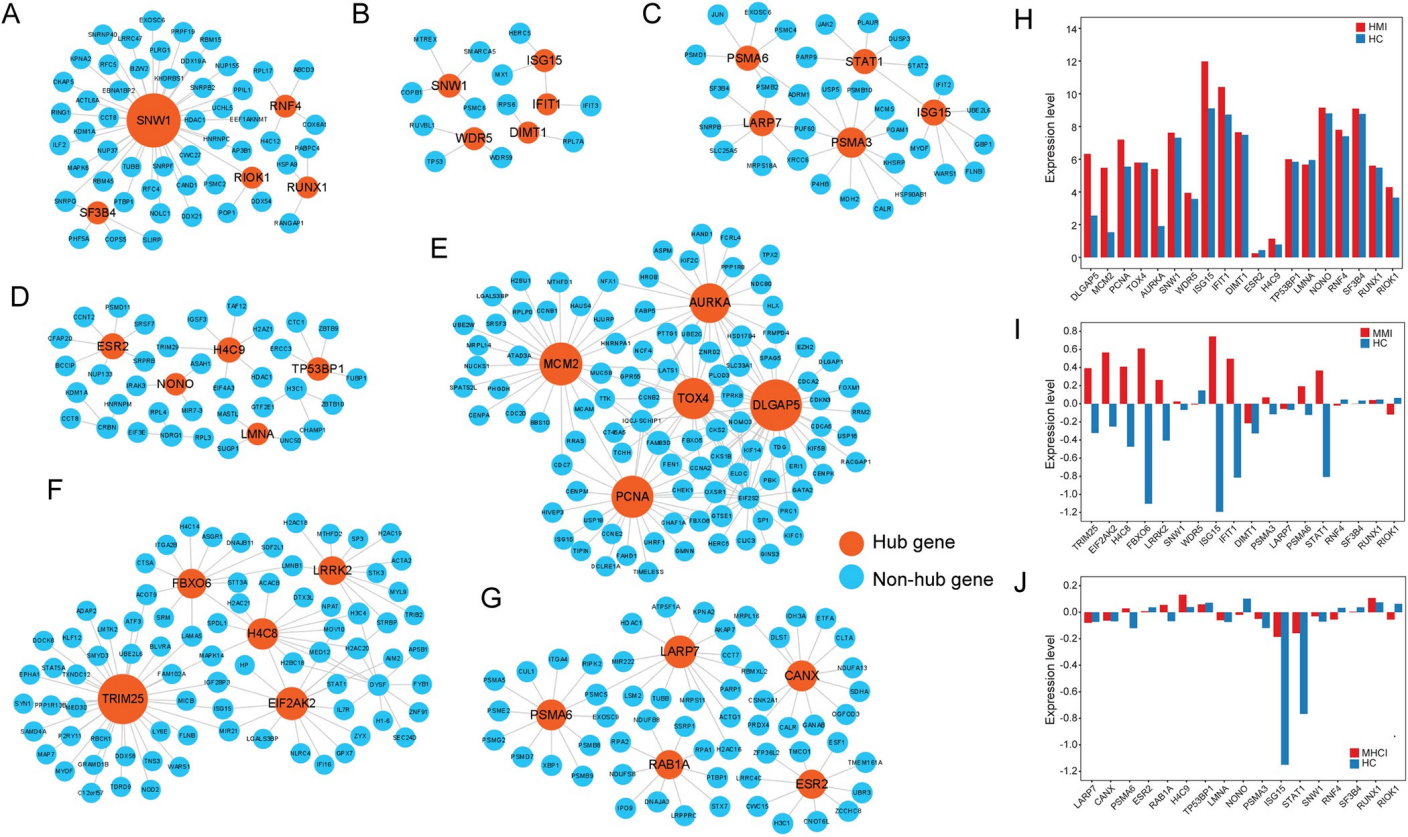
Analysis of hub genes in different subnetworks. (A) Hub genes in Common-P. (B) Hub genes in HMI-MMI-SP. (C) Hub genes in MMI-MHCI-SP. (D) Hub genes in HMI-MHCI-SP. (E) Hub genes in HMI-SP. (F) Hub genes in MMI-SP. (G) Hub genes in MHCI-SP. (H-J) Expression levels of hub genes in the disease and healthy states.

In this work, we defined the top five genes with highest degree values in each subclass of IDEN-P as the hub genes. We also compared the expression levels of these genes in the disease and normal states using three datasets (GSE29429, GSE69581 and GSE83456). In Common-P, the hub genes included *SNW1*, *SF3B4*, *RNF4*, *RIOK1* and *RUNX1* (Fig 9A). Generally, these genes had a wide range of functions and play critical roles in various biological processes (S7 Table). For instance, *RNF4* is highly involved in physiopathological processes and is useful for the repair of DNA damage [58,59]. *SF3B4* is a component of the splicing factor complex, and its abnormal expression may cause the dysregulation of alternative splicing [60,61]. Moreover, their expression levels showed small changes between different states (Fig 9H–9J), indicating that these genes were highly robust.

*ISG15* and *IFIT1* were two hub genes in HMI-MMI-SP (Fig 9B). *ISG15* could inhibit the replication and release of viruses [62]. As an interferon-induced protein, *IFIT1* plays a key role in immune responses [63]. The expression levels of *ISG15* and *IFIT1* were remarkably higher in the disease states, probably because these two interferon-related genes are abundantly expressed for strengthening immune defenses. Two proteasome-related proteins, namely *PSMA3* and *PSMA6*, were hub genes in MMI-MHCI-SP, and their higher expression in patients suggested that the host may utilize proteasomes to eliminate disease-related proteins (Fig 9C). Three hub genes (*TP53BP1*, *LMNA* and *NONO*) in HMI-MHCI-SP are involved in DNA damage repair and thus maintain the genome stability [64–66] (Fig 9D). Existing studies reported that overexpression of estrogen receptor (e.g. *ESR2*) could suppress viral replication [67]. Collectively, the hub genes shared by the two states showed the ability to enhance host immunity (S7 Table).

Most viruses invade hosts by regulating the cell cycle. The hub genes (*DLGAP5*, *MCM2*, *PCNA* and *AURKA*) in HMI-SP consistently encode cell cycle regulatory proteins that ensure proper chromosome segregation [68–72] (Fig 9E). Their higher expression levels in HMI were probably caused by HIV invasion (Fig 9H), resulting in abnormal cell cycle regulation and pathological forms of mitosis. Endoplasmic reticulum stress (ERS) is a critical cellular self-protective mechanism [73]. The hub gene *TRIM25* in MMI-SP can provide negative feedback regulation of ERS [74] (Fig 9F). We speculated that MTB may use *TRIM25* to respond to the ERS to accelerate the progression of TB disease. Moreover, *FBXO6* and *EIF2AK2* are also involved in ERS or cellular homeostatic responses [75,76]. The higher expression of these three genes in MMI supported the above speculation (Fig 9I). Transcriptional regulation controls the normal expression of genes, and disruption of this process may lead to disease [77]. In MHCI-SP, four hub genes (*LARP7*, *ESR2*, *CANX* and *RAB1A*) were reported to be involved in transcriptional regulation and translational processes [5,78–80] (Fig 9G). In contrast to the hub genes shared by two states, those specific to each state could facilitate pathogen infection (S7 Table).

### Drug repurposing based on gene pairs

**Fig 10:**
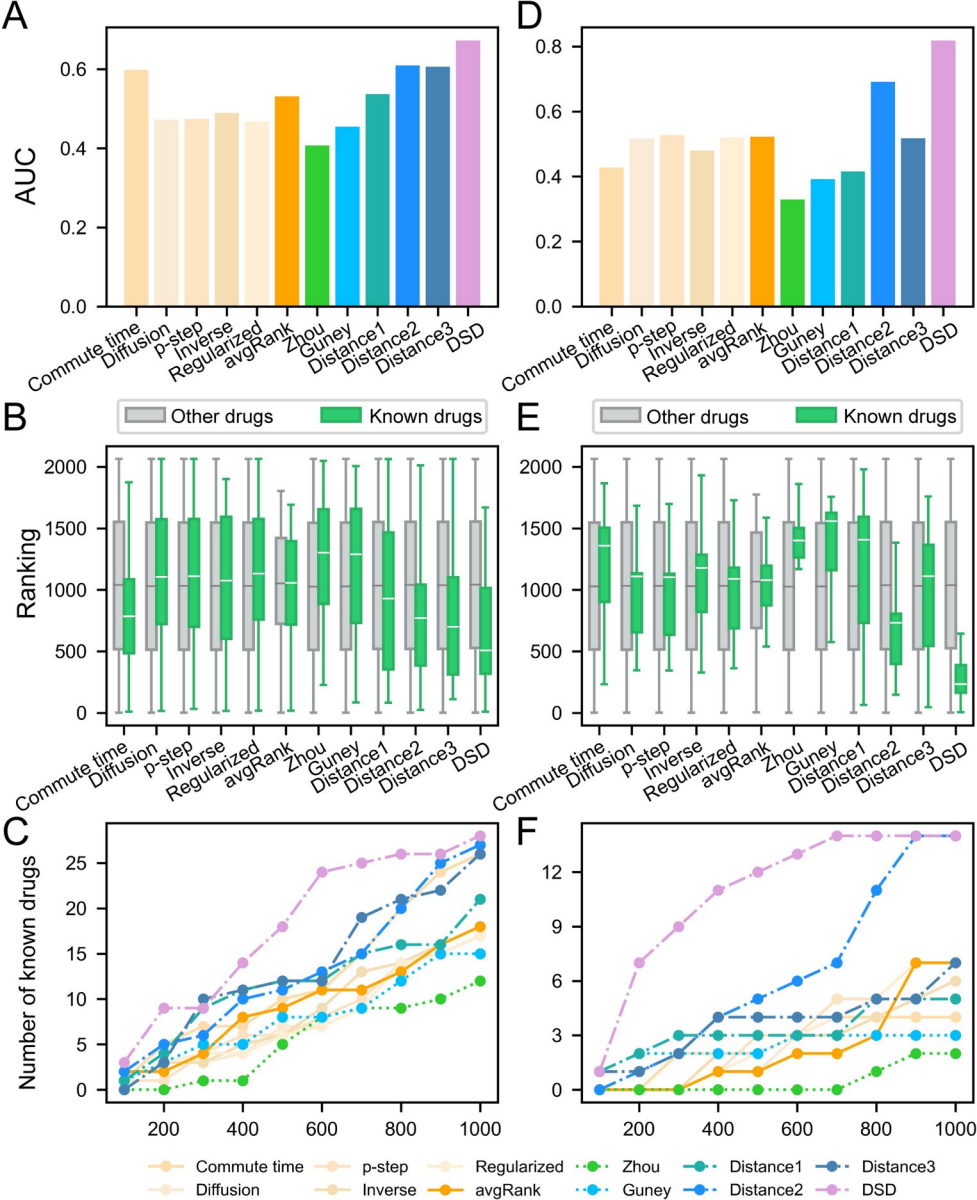
Comparison of our proximity measures and existing network-based drug repurposing methods. (A-C) Performance of different methods on known HIV drugs. (D-F) Performance of different methods on known TB drugs. Evaluation measures include the AUC for identifying known drugs, rankings of known drugs and other drugs, and numbers of known drugs appearing in the top K drugs. These algorithms include our gene pair-based distance measures (distance1, distance2 and distance3), disease gene-based distance measures (Guney’s distance and Zhou’s distance), graph kernel methods (commute time, diffusion, p-step, regularized Laplacian, inverse cosine and avgRank), and diffusion state distance methods (DSD).

Here, we evaluated the three interaction-based proximity metrics proposed in this work. Furthermore, we compared our algorithms with existing network-based drug repurposing methods, including the Guney’s distance [19,20], Zhou’s distance [17], diffusion state distance (DSD) methods [19], and graph kernel-based methods [18]. All competing algorithms explored the relationship between drug targets and disease genes rather than gene pairs. The kernel methods were implemented using in-house scripts, and the other competing approaches were implemented using the codes on GitHub. Among the 2065 approved drugs collected in this study, 38 HIV- and 15 TB-related drugs could be considered as positive samples (S2 Data). If one algorithm assigns the higher rankings for known drugs, this method may provide more effective predictions. The evaluation measures (e.g. AUC, recall and precision at the top rankings) are the same as those used by Santos et al.’s work [18]. As shown in Fig 10 and S8 and S9 Tables, our distance measures performed more favorably than disease gene-based proximity metrics (i.e. Guney’s distance and Zhou’s distance), suggesting that incorporation of disease-related interactions is indeed useful for drug repurposing. Our methods also showed advantages over other network-based algorithms except the DSD method. Among our three measures, the distance *d*_2_(*P*,*T*) achieved generally better performance than the distance *d*_1_(*P*,*T*) and the combined distance *d*_3_(*P*,*T*). This may be because fixing the bigger gene set (e.g. the PPI set) and computing the shortest distance to the genes in the smaller set (e.g. the drug target set) could more effectively reflect the proximity. Based on the analysis, the distance *d*_2_(*P*,*T*) was finally selected for drug repurposing in this work.

By observing the distribution of standardized proximity metrics, we considered that the drugs with a Z-score less than -1.036 may be effective against pathogen infections. We thus obtained 178, 150 and 169 drugs (approximately the top 8% of all drugs) for MMI, HMI and MHCI, respectively (S2 Data). Then, we compared the up- and down-regulated DEGs of each disease state against the drug-induced gene expression profiles in CMAP. The GSEA score provided by CMAP could be used as an auxiliary indication to validate the above candidates (a negative score was preferred). Moreover, we ensured that each candidate has anti-pathogen evidences by manually checking the literature. Finally, we retained drugs having fewer side effects and excluded nutritional drugs, metal drugs (e.g. copper and zinc) and radioligand diagnostic agents. According to the above criteria, 10, 10 and 11 drugs were chosen for MMI, HMI and MHCI, respectively.

#### Repurposable drug candidates for MMI

**Fig 11:**
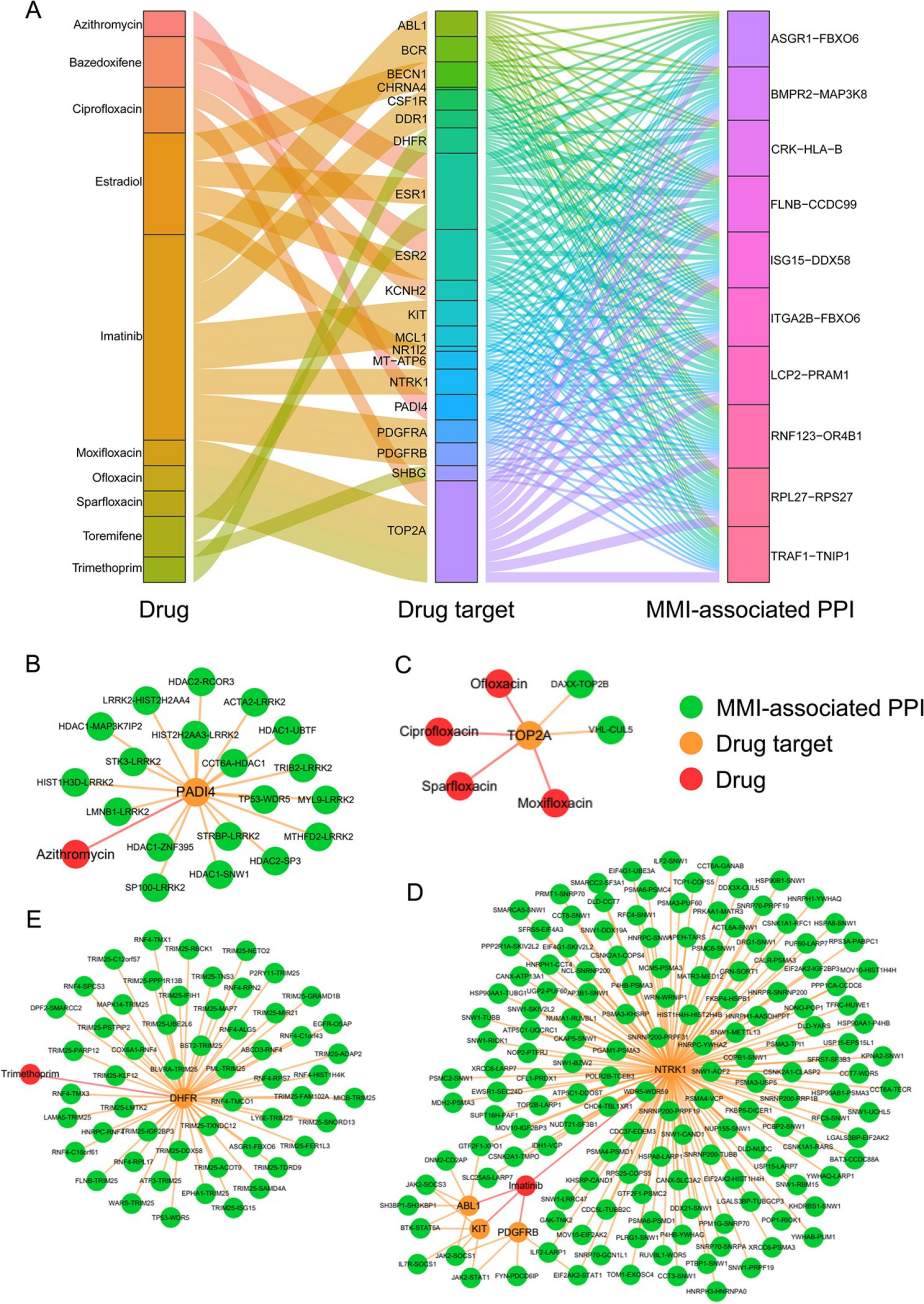
Drug-target-PPI associations for MMI. (A) Relationship between anti-MMI drug candidates and MMI-associated PPIs. Top 10 PPIs with the highest number of shortest links to drug targets are shown (one PPI may correspond to multiple targets). (B) Closest PPIs regulated by azithromycin. (C) Closest PPIs regulated by ciprofloxacin, moxifloxacin, ofloxacin and sparfloxacin. (D) Closest PPIs regulated by imatinib. (E) Closest PPIs regulated by trimethoprim.

Among the 10 identified drugs, several candidates have been applied to the treatment of bacterial diseases (Fig 11A and S10 Table). As a macrolide antibiotic, for instance, azithromycin could prevent various bacterial infections [81] (Fig 11B). This drug blocks bacterial protein synthesis by binding to the 50S ribosomal subunit [82]. Moxifloxacin, a broad-spectrum fluoroquinolone antibiotic, functions by inhibiting topoisomerases II and IV, therefore interfering with bacterial DNA replication, transcription and repair [83] (Fig 11C). Additionally, ciprofloxacin, sparfloxacin and ofloxacin are also fluoroquinolones and may exhibit similar anti-TB effects. All these four drugs could mediate two gene pairs, *TOP2B*-*DAXX* and *VHL*-*CUL5* (Fig 11C). Imatinib is a tyrosine kinase inhibitor for the treatment of malignant tumors. This agent inhibits tyrosine kinases *ABL1* and *ABL2*, remarkably reducing the bacterial load in MTB-infected mice [84,85]. Our analysis showed that imatinib may adopt drug targets *KIT* and *PDGFRB* to regulate *JAK2*-*STAT1*, of which *STAT1* is a key factor in the interferon signaling pathway to resist MTB infection [86] (Fig 11D). Trimethoprim is an anti-folate antibiotic that suppresses bacterial dihydrofolate reductase (*DHFR*), thereby blocking bacterial DNA synthesis and ultimately inhibiting bacterial survival (Fig 11E). Several studies showed that trimethoprim combined with sulfamethoxazole has strong antibacterial activity against MTB in clinical treatment [87–89].

#### Repurposable drug candidates for HMI

Our algorithm identified 10 potential anti-HMI agents (S10A Fig and S11 Table). Raloxifene is a second-generation nonsteroidal selective estrogen receptor modulator that could counter virus infections [90–92]. This compound could use three targets (*ESR1*, *ESR2* and *SERPINB9*) to regulate *MCM2*-*TUBB2A*, of which *MCM2* can effectively block HIV-1 infection of macrophages by inhibiting viral DNA synthesis [70,93] (S10B Fig). Angiotensin receptor blockers (ARBs) have been reported to combat viral infections (e.g. human coronaviruses) [94,95]. As a representative ARB, irbesartan is mainly used for treating hypertension and diabetic nephropathy. This candidate is highly associated with HIV-related PPIs, such as *NOLC1-SNW1* and *SF3B4-COPS5* [96,97] (S10C Fig). In principle, disrupting the key stages of the HIV life cycle could resist HIV infection. Six drug candidates (i.e. fludarabine, clofarabine, cladribine, gemcitabine, topotecan and irinotecan) could inhibit DNA or RNA production, thereby blocking the transcription or translation process of HIV [98–100]. Estradiol is a sex hormone having anti-inflammatory properties, which could protect nerve cells from injuries and chronic diseases, including neuroAIDS [101,102]. In HIV-1 infected cells, estradiol may inhibit HIV-1 replication by directly altering its transcriptional activation [103,104]. Through targets *ESR1*, *ESR2* and *NR1I2*, estradiol could mediate *NCOA6-NCOA2*, of which *NCOA6* regulates the expression of HIV-1 long terminal repeats, and *NCOA2* serves as a coactivator for HIV-1 Tat proteins [105,106] (S10D Fig).

#### Repurposable drug candidates for MHCI

The co-infection is not a simple superposition of two mono-infections, and there may be a more complex pathogenesis behind MHCI. Therefore, we should develop rational drugs for the specific pathogenic mechanisms of co-infection in addition to inhibiting the mono-infection of the two pathogens. Among the 11 identified drugs, ciprofloxacin, trimethoprim and toremifene were proposed to resist MTB infection, while topotecan, irinotecan and irbesartan were identified to prevent HIV infection (S11A Fig and S12 Table). Moreover, we obtained another three drugs that were specific to MHCI. Vinorelbine and vinblastine are both anti-mitotic chemotherapeutic agents that block microtubule polymerization to stop cell division in mitosis [107,108] (S11B Fig). These two drugs have been applied to the treatment of HIV-1 patients. The inflammatory response caused by pathogen infections is a primary cause of tissue damage and death [109]. Quercetin, widely distributed in fruits and vegetables, is a flavonoid compound with anti-oxidant and anti-inflammatory effects [110] (S11C Fig). Quercetin could function as the inhibitor of HIV reverse transcriptase and suppress the activities of various cellular DNA and RNA polymerases [111]. In vitro cell assays also showed that quercetin exhibits modest anti-HIV activities [112]. Additionally, extensive studies reported that quercetin has certain efficacy against TB [113–115]. For example, quercetin could inhibit a variety of enzymes (e.g. isocitrate lyase and hyaluronidase) that are essential for the growth and survival of MTB [116]. Our predictions implied that it could also have a host-directed mechanism of action and is a promising drug for the treatment of co-infection.

## Discussion

TB and AIDS are the two most lethal infectious diseases which are closely interconnected in the world. Although some efforts have been devoted to exploring the pathogeneses of MMI, HMI and MHCI, the similarities and specificities of the host responses to these infections have not been systematically compared and analyzed. To this end, our study designed a computational framework named IDEN to construct differential expression networks for the three disease states by combining differential and non-differential interactions, which were generated by the DCA and DEA methods, respectively. This framework identified 1007 genes and 911 gene pairs for MMI, 1266 genes and 1314 gene pairs for HMI and 1576 genes and 1363 gene pairs for MHCI. The identified genes were significantly enriched in known host targets of MTB and HIV as well as in functionally important gene sets and could be adopted as signatures to discriminate between disease and normal samples. We then compared the three networks from different viewpoints. At the genomic level, MMI-G and MHCI-G had the highest enrichment ratio on a greater number of chromosomes compared to HMI-G, and a similar trend was shown for the associated SNPs, dSNPs and disease-causing genes in MMI. Moreover, we found that genes shared by more statuses tended to be more evolutionarily conserved, posttranslationally modified and topologically important. Gene expression analyses showed that Common-G showed the highest expression level and HMI-SG held the lowest expression level. Regarding the gene pairs, HMI-SP yielded higher expression correlations than the other specific groups, while the overlapping pairs associated with co-infections had significantly lower correlations in the disease states. The patterns of expression correlation changes of Common-P revealed that MHCI may be more similar to MMI. Gene enrichment analyses exhibited that MMI-G and MHCI-G shared more pathways and biological processes, further illustrating that the pathogenic mechanism of TB might play a dominant role in the progression of MHCI. Functional analyses of the hub nodes in subnetworks suggested that the hub genes specific to each disease state may promote pathogen infections, while the hub genes shared by two disease states may enhance immune responses. Regarding drug repurposing, the network-based strategy has been successfully applied to this field, but existing works only considered the relationship between known drug targets and disease-related proteins rather than disease-related PPIs. We thus improved the existing proximity measure by focusing on gene pairs detected in this work. Finally, approximately ten reusable drugs were identified for each disease state, which may provide new clues to the therapy for relevant diseases.

Despite the results derived from this study, there is still room for improvement in the future work. First, the available datasets of relevant blood expression profiles were relatively limited. Although we designed the customized IDEN method for the current datasets, this framework could be improved by integrating other transcriptomic data (e.g. RNA-seq). Additional datasets could further validate the reliability of current results. Second, we only used the interactions from HIPPIE to build the reference PPI network of IDEN, which may lead to false positives in the identified gene pairs. To solve this problem, we could use a more rigorous threshold for confidence scores in HIPPIE or combine other PPI resources (e.g. PDB database) to strengthen the quality of human PPI network. Third, we used consistent PCC thresholds in the second and third filtering steps to detect DIs for different disease states (S2 Fig), which may lose gene pairs specific to a certain state. In future we could design adaptive thresholds for each state to retrieve DIs. Fourth, the drug-target network for drug repurposing only included records from DrugBank and TTD. Integration of more drug-related resources (e.g. PharmGKB, ChEMBL and BindingDB databases) may improve the coverage of drug-target interactions, thereby providing more insights into reusable drugs. Fifth, although most proposed drugs in this work have been supported by comprehensive evidences, all drugs must undergo rigorous randomized clinical trials before they can be used to treat patients. Sixth, in addition to AIDS, other diseases (e.g. diabetes and hypertension) were interconnected with TB. We could conduct the similar comparison and analysis for other complex disease states. In conclusion, we provide an integrative framework for the identification of important gene pairs and reusable drugs for the infections caused by MTB and/or HIV, which may help deepen our understanding of the mechanisms underlying the interactions between the host and pathogens.

## Supporting information

S1 Fig Normalized expression profiles of randomly selected samples in each dataset. (PDF)

S2 Fig Process of extracting DIs by the DCA method. (A) Extraction of DIs for HMI. (B) Extraction of DIs for MMI. (C) Extraction of DIs for MHCI.
(PDF)

S3 Fig Samples in GSE69581 and GSE83456 datasets before and after removal of batch effects. (A) Normalized expression profiles of samples. (B) Principal component analysis of samples. Left: results before batch effect removal, middle: results after batch effect removal and right: results after quantile normalization.
(PDF)

S4 Fig Volcano plot of DEGs for three disease states. (A) DEGs of HMI. (B) DEGs of MMI. (C) DEGs of MHCI.
(PDF)

S5 Fig Classification of genes and gene pairs identified by IDEN framework. (A) Genes identified by the IDEN (IDEN-G). (B) Gene pairs identified by the IDEN (IDEN-P). (C) Seven subclasses of IDEN-G or IDEN-P.
(PDF)

S6 Fig Genomic features of disease-related genes. (A) Chromosomal distribution of detected genes. (B) Chromosomal distribution of SNP density of detected genes. (C) Proportion of disease-related SNPs among total SNPs of detected genes. (D) Proportion of disease-causing genes among detected genes.
(PDF)

S7 Fig Chromosomal distance of disease-related genes in different subclasses. (PDF)

S8 Fig Expression level of disease-related genes in different groups. (PDF)

S9 Fig Gene ontology analysis of disease-related genes. (A) MMI-G. (B) HMI-G. (C) MHCI-G. (D) Common-G.
(PDF)

S10 Fig Drug-target-PPI associations for HMI. (A) Relationship between anti-HMI drug candidates and HMI-associated PPIs. Top 10 PPIs with the highest number of shortest links to drug targets are shown (one PPI may correspond to multiple targets). (B) Closest PPIs regulated by raloxifene. (C) Closest PPIs regulated by irbesartan. (D) Closest PPIs regulated by estradiol.
(PDF)

S11 Fig Drug-target-PPI associations for MHCI. (A) Relationship between anti-MHCI drug candidates and MHCI-associated PPIs. Top 10 PPIs with the highest number of shortest links to drug targets are shown (one PPI may correspond to multiple targets). (B) Closest PPIs regulated by vinblastine and vinorelbine. (C) Closest PPIs regulated by quercetin.
(PDF)

S1 Table Gene expression profile datasets used in this study. (PDF)

S2 Table Overlap between identified genes and existing resources. (PDF)

S3 Table Number of IDEN genes enriched in functionally important gene sets. (PDF)

S4 Table Number of subclass genes enriched in functionally important gene sets. (PDF)

S5 Table Performance of genes and gene pairs for classification of different samples. (PDF)

S6 Table AUC values achieved by different gene signatures. (PDF)

S7 Table Biological functions of hub genes in different subnetworks. (PDF)

S8 Table Recall and precision at the top K drugs of different methods for HIV drugs. (PDF)

S9 Table Recall and precision at the top K drugs of different methods for TB drugs. (PDF)

S10 Table Repurposed anti-MMI drug candidates. (PDF)

S11 Table Repurposed anti-HMI drug candidates. (PDF)

S12 Table Repurposed anti-MHCI drug candidates. (PDF)

S1 Data Original data used for generating figures. (XLSX)

S2 Data Original data used for drug repurposing. (XLSX)
