# Supplementary figures and images for: Systematic comparison of differential expression networks in MTB mono-, HIV mono- and MTB/HIV co-infections for drug repurposing
Source: PLoS Comput Biol. 2022 Dec 19;18(12):e1010744. doi: 10.1371/journal.pcbi.1010744 (PMC9810203; doi:10.1371/journal.pcbi.1010744)

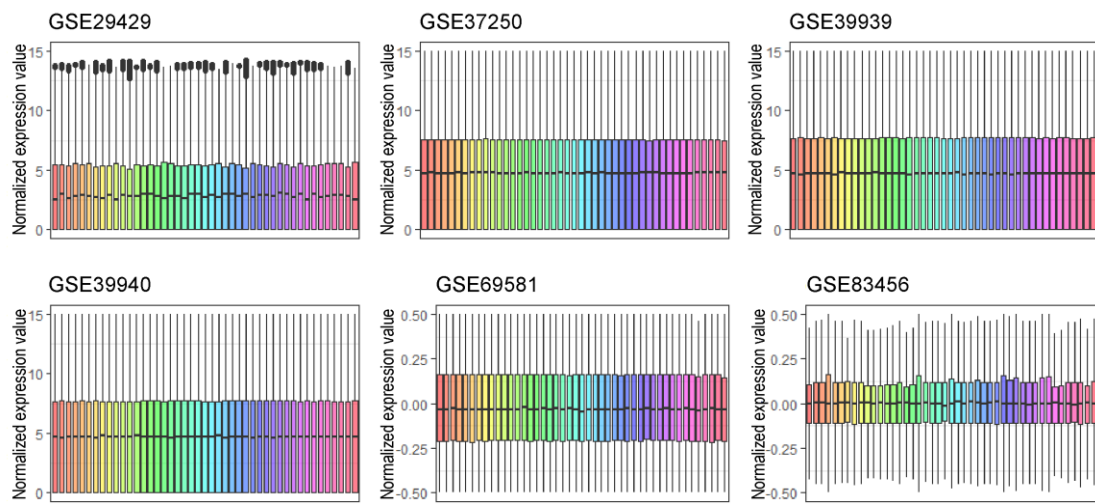

**S1 Fig. Normalized expression profiles of randomly selected samples in each dataset.**

Supplement: S1 Fig — (PDF) [file pcbi.1010744.s001.pdf]

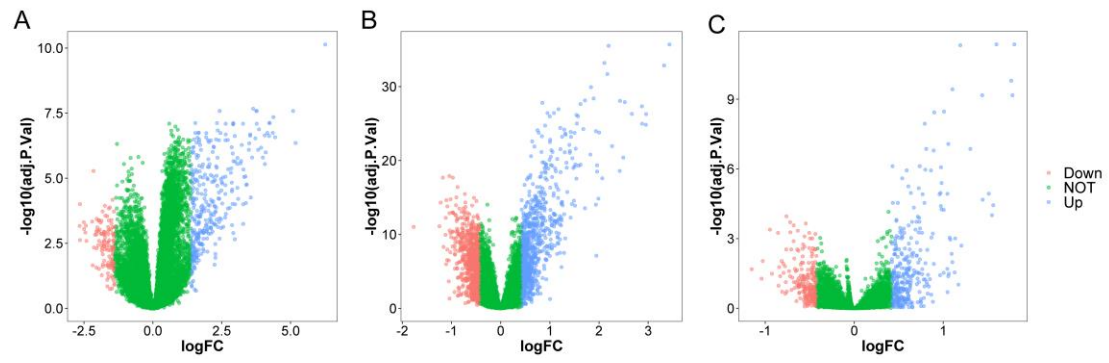

**S4 Fig. Volcano plot of DEGs for three disease states.** (A) DEGs of HMI. (B) DEGs of MMI. (C) DEGs of MHCI.

Supplement: S4 Fig — (A) DEGs of HMI. (B) DEGs of MMI. (C) DEGs of MHCI. (PDF) [file pcbi.1010744.s004.pdf]

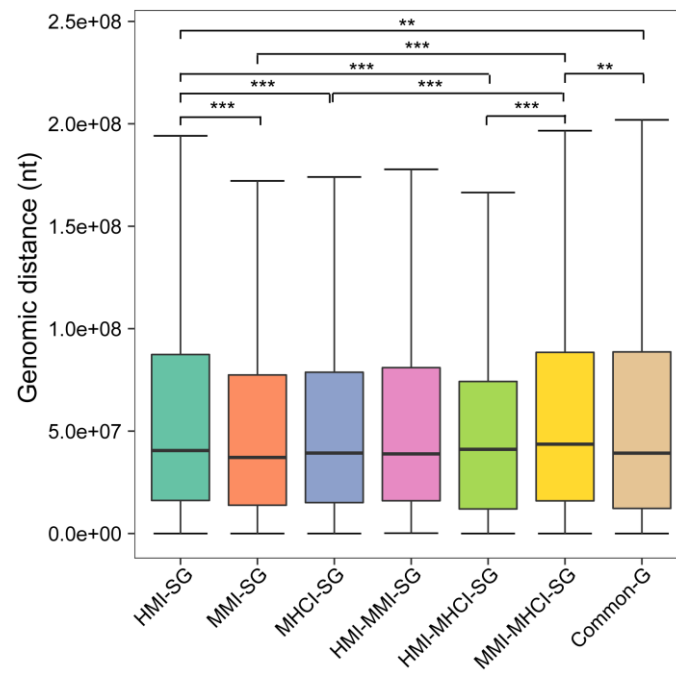

**S7 Fig. Chromosomal distance of disease-related genes in different subclasses.**

Supplement: S7 Fig — (PDF) [file pcbi.1010744.s007.pdf]

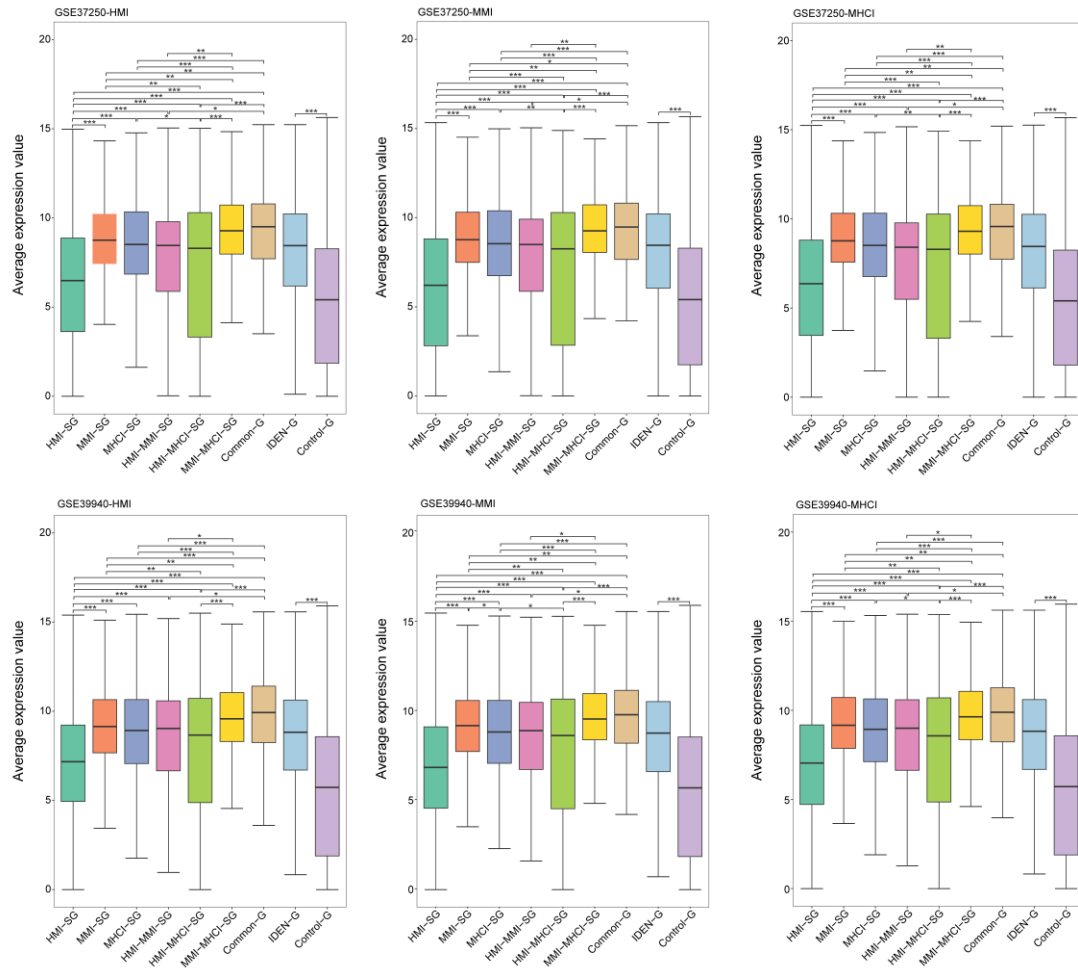

**S8 Fig. Expression level of disease-related genes in different groups.**

Supplement: S8 Fig — (PDF) [file pcbi.1010744.s008.pdf]

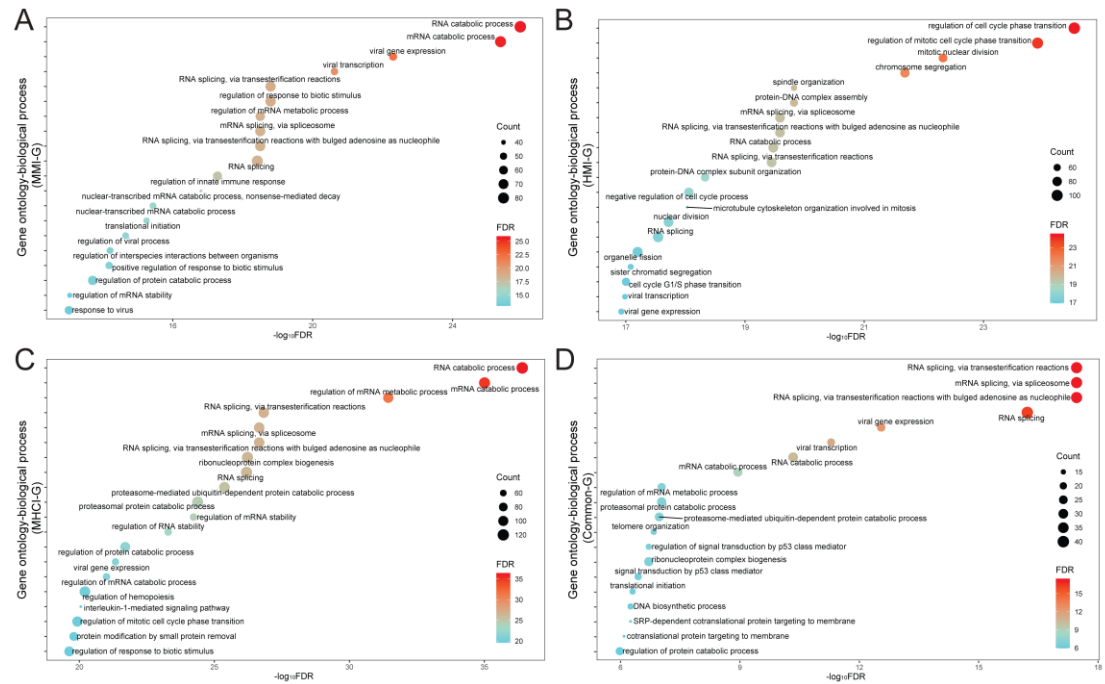

**S9 Fig. Gene ontology analysis of disease-related genes. (A) MMI-G. (B) HMI-G. (C) MHC-G. (D) Common-G.**

Supplement: S9 Fig — (A) MMI-G. (B) HMI-G. (C) MHCI-G. (D) Common-G. (PDF) [file pcbi.1010744.s009.pdf]
