# Supplementary material for: Systematic comparison of differential expression networks in MTB mono-, HIV mono- and MTB/HIV co-infections for drug repurposing
Source: PLoS Comput Biol. 2022 Dec 19;18(12):e1010744. doi: 10.1371/journal.pcbi.1010744 (PMC9810203; doi:10.1371/journal.pcbi.1010744)
Supplement: S2 Fig — (A) Extraction of DIs for HMI. (B) Extraction of DIs for MMI. (C) Extraction of DIs for MHCI. (PDF) [file pcbi.1010744.s002.pdf]

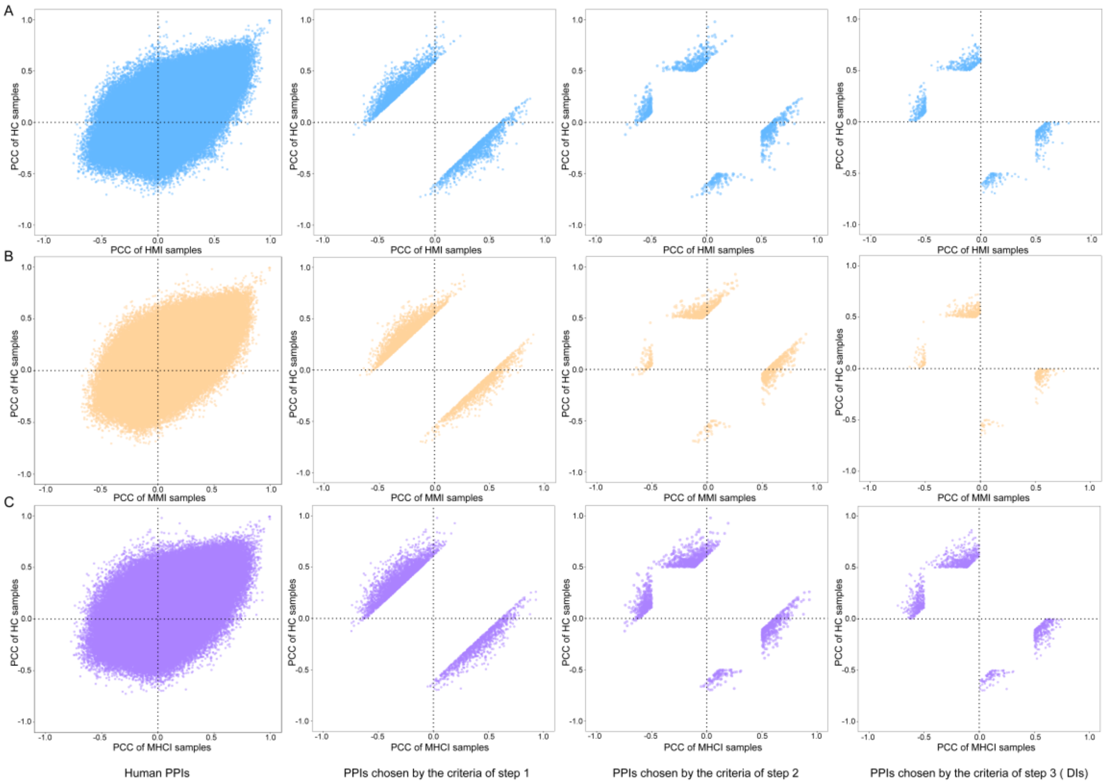

S2 Fig. Process of extracting DIs by the DCA method. (A) Extraction of DIs for HMI. (B) Extraction of DIs for MMI. (C) Extraction of DIs for MHCI.
