# Supplementary material for: Systematic comparison of differential expression networks in MTB mono-, HIV mono- and MTB/HIV co-infections for drug repurposing
Source: PLoS Comput Biol. 2022 Dec 19;18(12):e1010744. doi: 10.1371/journal.pcbi.1010744 (PMC9810203; doi:10.1371/journal.pcbi.1010744)
Supplement: S3 Fig — (A) Normalized expression profiles of samples. (B) Principal component analysis of samples. Left: results before batch effect removal, middle: results after batch effect removal and right: results after quantile normalization. (PDF) [file pcbi.1010744.s003.pdf]

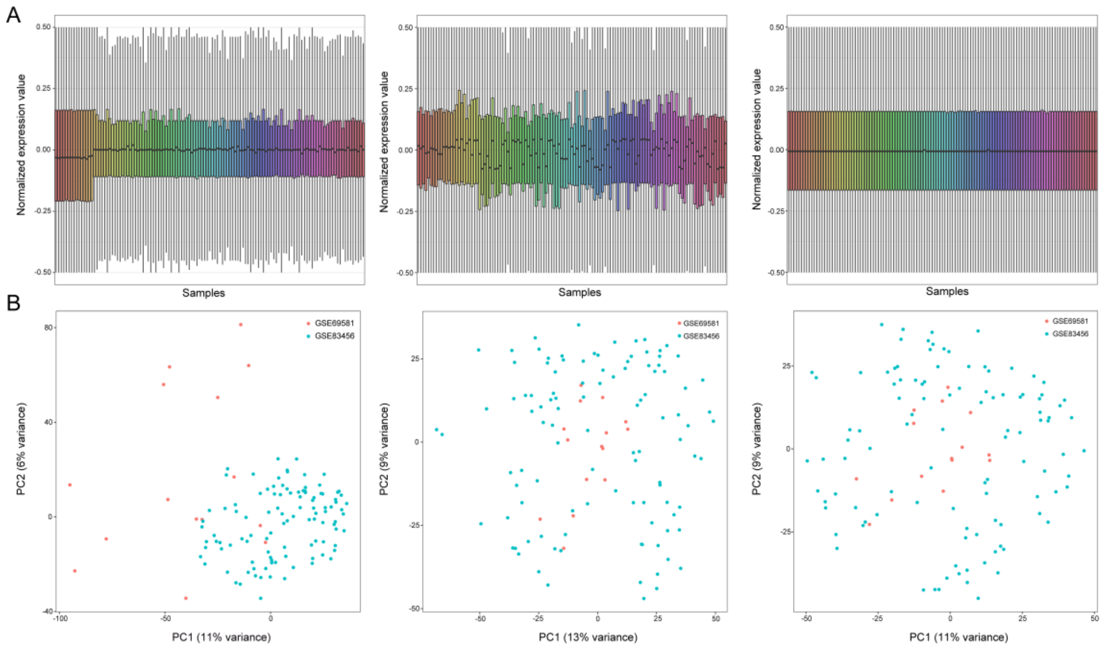

S3 Fig. Samples in GSE69581 and GSE83456 datasets before and after removal of batch effects. (A) Normalized expression profiles of samples. (B) Principal component analysis of samples. Left: results before batch effect removal, middle: results after batch effect removal and right: results after quantile normalization.
