# Supplementary material for: Systematic comparison of differential expression networks in MTB mono-, HIV mono- and MTB/HIV co-infections for drug repurposing
Source: PLoS Comput Biol. 2022 Dec 19;18(12):e1010744. doi: 10.1371/journal.pcbi.1010744 (PMC9810203; doi:10.1371/journal.pcbi.1010744)
Supplement: S5 Fig — (A) Genes identified by the IDEN (IDEN-G). (B) Gene pairs identified by the IDEN (IDEN-P). (C) Seven subclasses of IDEN-G or IDEN-P. (PDF) [file pcbi.1010744.s005.pdf]

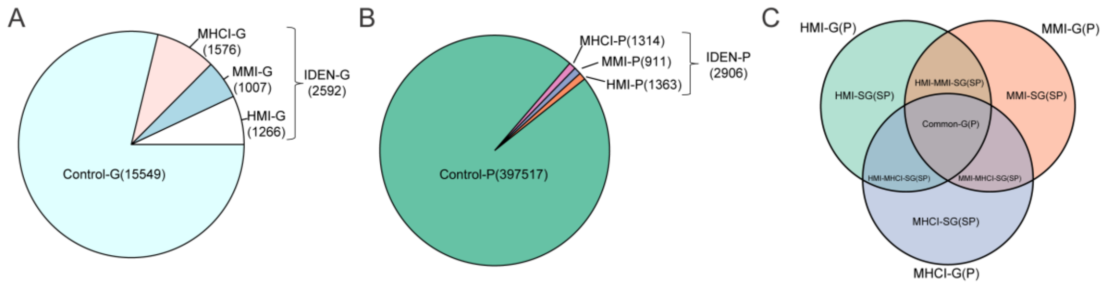

S5 Fig. Classification of genes and gene pairs identified by IDEN framework. (A) Genes identified by the IDEN (IDEN-G). (B) Gene pairs identified by the IDEN (IDEN-P). (C) Seven subclasses of IDEN-G or IDEN-P.
