# Supplementary material for: Systematic comparison of differential expression networks in MTB mono-, HIV mono- and MTB/HIV co-infections for drug repurposing
Source: PLoS Comput Biol. 2022 Dec 19;18(12):e1010744. doi: 10.1371/journal.pcbi.1010744 (PMC9810203; doi:10.1371/journal.pcbi.1010744)
Supplement: S6 Fig — (A) Chromosomal distribution of detected genes. (B) Chromosomal distribution of SNP density of detected genes. (C) Proportion of disease-related SNPs among total SNPs of detected genes. (D) Proportion of disease-causing genes among detected genes. (PDF) [file pcbi.1010744.s006.pdf]

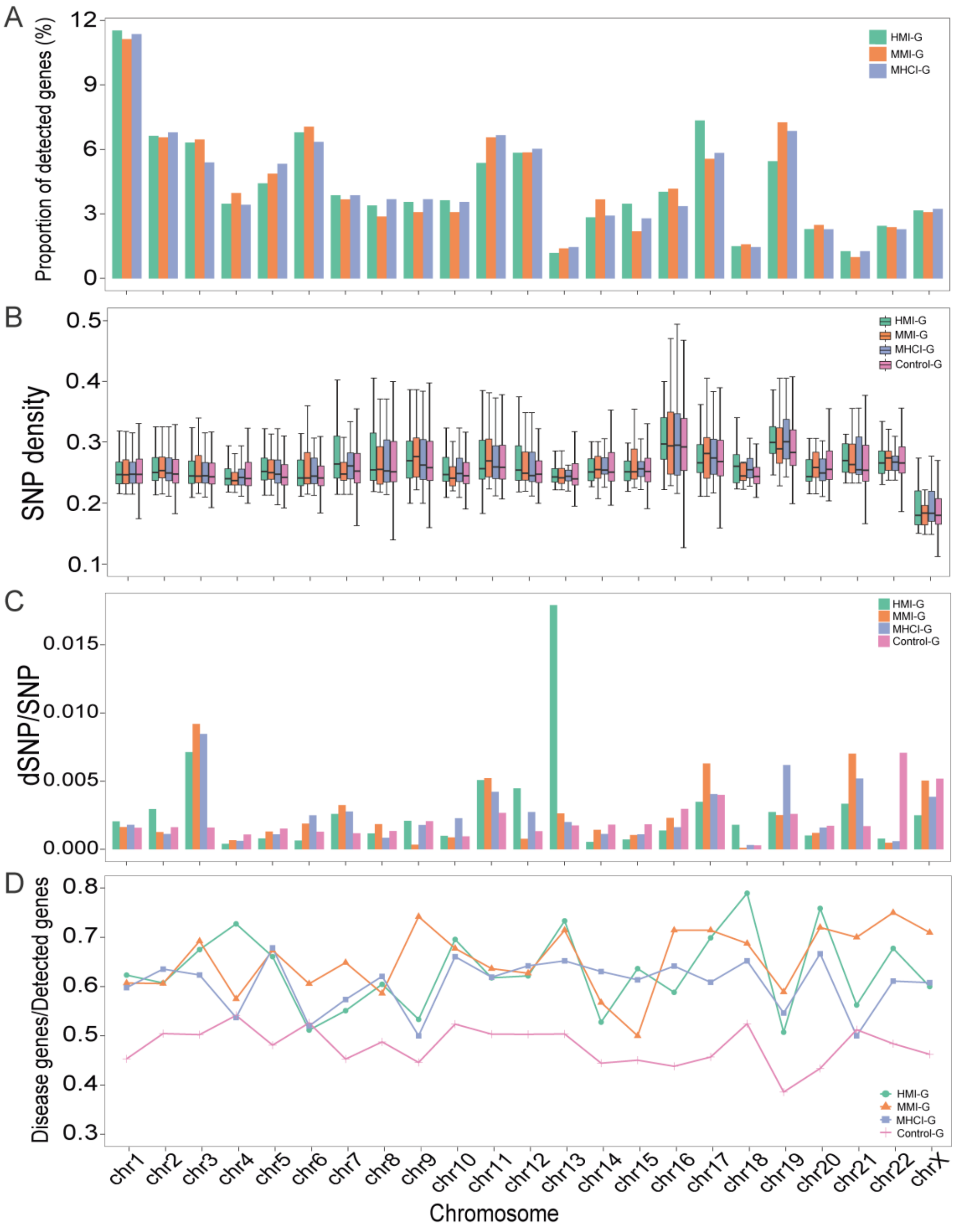


**S6 Fig. Genomic features of disease-related genes.** (A) Chromosomal distribution of detected genes. (B) Chromosomal distribution of SNP density of detected genes. (C) Proportion of disease-related SNPs among total SNPs of detected genes. (D) Proportion of disease-causing genes among detected genes.
