# Supplementary material for: Systematic comparison of differential expression networks in MTB mono-, HIV mono- and MTB/HIV co-infections for drug repurposing
Source: PLoS Comput Biol. 2022 Dec 19;18(12):e1010744. doi: 10.1371/journal.pcbi.1010744 (PMC9810203; doi:10.1371/journal.pcbi.1010744)
Supplement: S10 Fig — (A) Relationship between anti-HMI drug candidates and HMI-associated PPIs. Top 10 PPIs with the highest number of shortest links to drug targets are shown (one PPI may correspond to multiple targets). (B) Closest PPIs regulated by raloxifene. (C) Closest PPIs regulated by irbesartan. (D) Closest PPIs regulated by estradiol. (PDF) [file pcbi.1010744.s010.pdf]

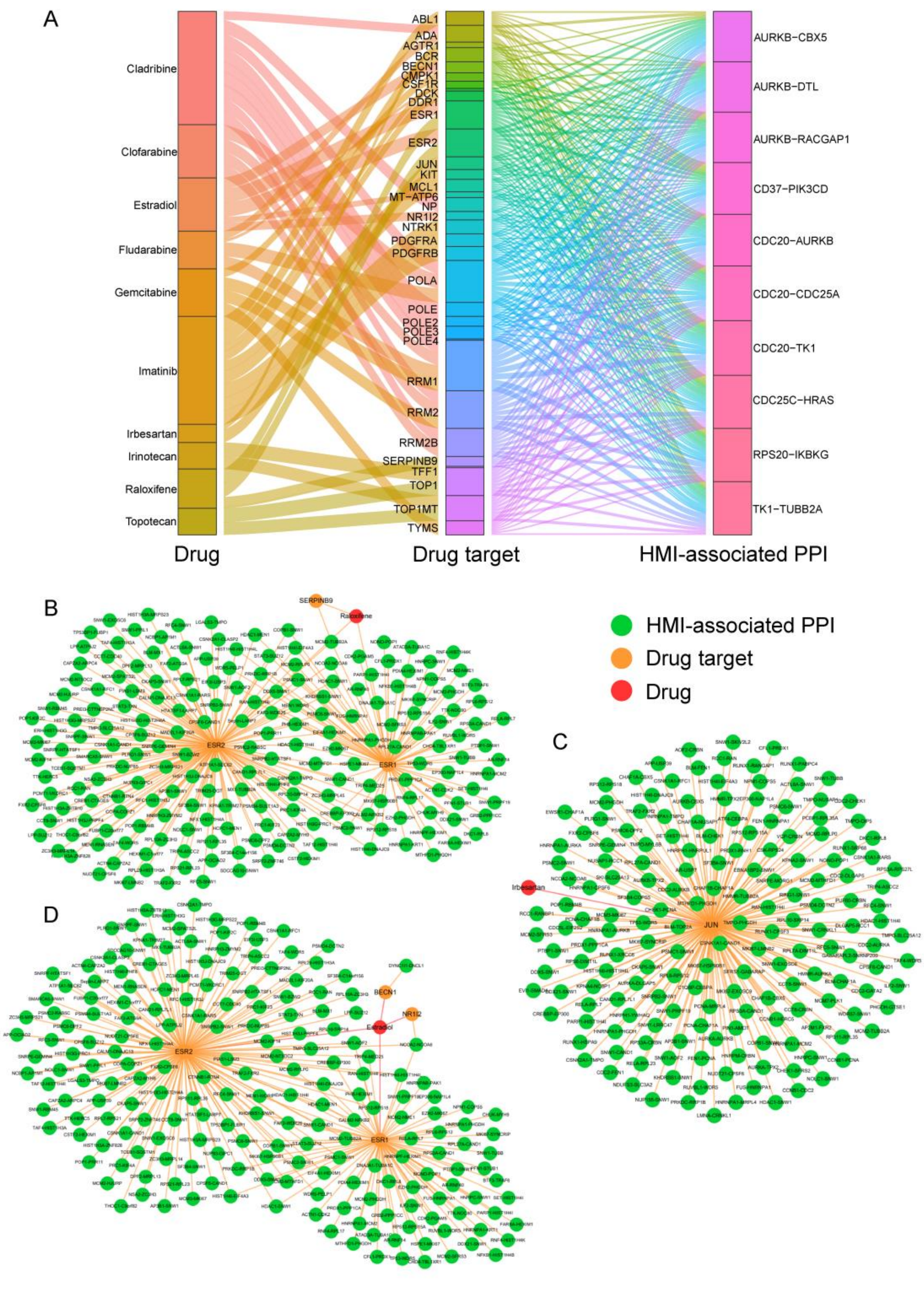

S10 Fig. Drug-target-PPI associations for HMI. (A) Relationship between anti-HMI drug candidates and HMI-associated PPIs. Top 10 PPIs with the highest number of shortest links to drug targets are shown (one PPI may correspond to multiple targets). (B) Closest PPIs regulated by raloxifene. (C) Closest PPIs regulated by irbesartan. (D) Closest PPIs regulated by estradiol.
