# Supplementary material for: Systematic comparison of differential expression networks in MTB mono-, HIV mono- and MTB/HIV co-infections for drug repurposing
Source: PLoS Comput Biol. 2022 Dec 19;18(12):e1010744. doi: 10.1371/journal.pcbi.1010744 (PMC9810203; doi:10.1371/journal.pcbi.1010744)
Supplement: S11 Fig — (A) Relationship between anti-MHCI drug candidates and MHCI-associated PPIs. Top 10 PPIs with the highest number of shortest links to drug targets are shown (one PPI may correspond to multiple targets). (B) Closest PPIs regulated by vinblastine and vinorelbine. (C) Closest PPIs regulated by quercetin. (PDF) [file pcbi.1010744.s011.pdf]

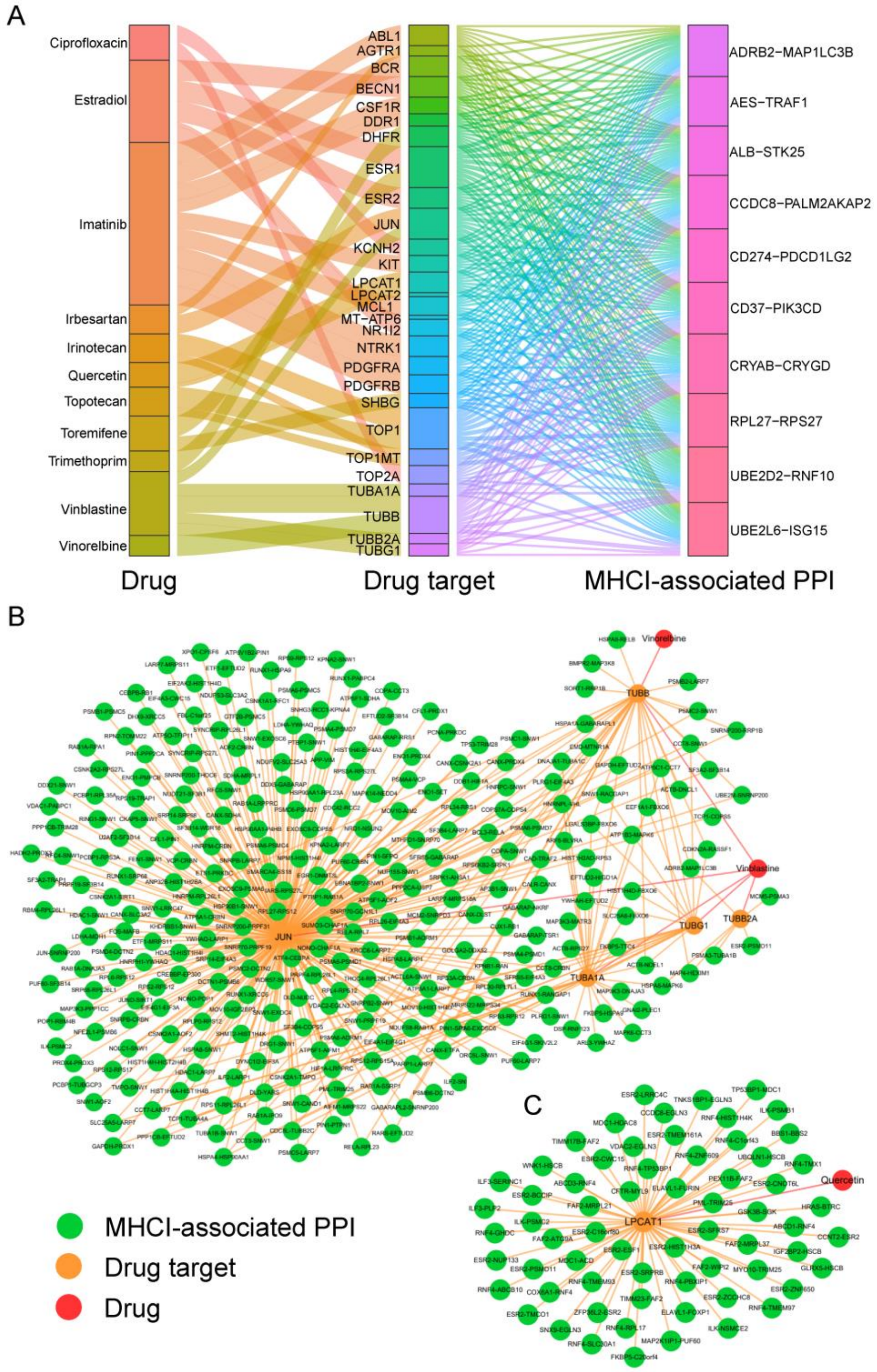

S11 Fig. Drug-target-PPI associations for MHCI. (A) Relationship between anti-MHCI drug candidates and MHCI-associated PPIs. Top 10 PPIs with the highest number of shortest links to drug targets are shown (one PPI may correspond to multiple targets). (B) Closest PPIs regulated by vinblastine and vinorelbine. (C) Closest PPIs regulated by quercetin.
