# Supplementary material for: Systematic comparison of differential expression networks in MTB mono-, HIV mono- and MTB/HIV co-infections for drug repurposing
Source: PLoS Comput Biol. 2022 Dec 19;18(12):e1010744. doi: 10.1371/journal.pcbi.1010744 (PMC9810203; doi:10.1371/journal.pcbi.1010744)
Supplement: S1 Table — (PDF) [file pcbi.1010744.s012.pdf]

**S1 Table. Gene expression profile datasets used in this study**

| Dataset | No. of samples | Reference |
|---|---|---|
| GSE29429 | 30 HMI and 17 HC | -- |
| GSE37250 | 97 MMI, 98 MHCI and 92 HMI | [1] |
| GSE39939 | 25 MMI, 10 MHCI and 26 HMI | [2] |
| GSE39940 | 70 MMI, 41 MHCI and 66 HMI | [2] |
| GSE69581 | 15 MHCI | [3] |
| GSE83456 | 45 MMI and 61 HC | [4] |

HMI refers to HIV mono-infected samples, MMI refers to MTB mono-infected samples, MHCI refers to co-infected samples, and HC refers to healthy samples.

## References

1. Kaforou M, Wright VJ, Oni T, French N, Anderson ST, Bangani N, et al. Detection of tuberculosis in HIV-infected and -uninfected African adults using whole blood RNA expression signatures: a case-control study. PLoS Med. 2013;10(10):e1001538.
2. Anderson ST, Kaforou M, Brent AJ, Wright VJ, Banwell CM, Chagaluka G, et al. Diagnosis of childhood tuberculosis and host RNA expression in Africa. N Engl J Med. 2014;370(18):1712-23.
3. Esmail H, Lai RP, Lesosky M, Wilkinson KA, Graham CM, Horswell S, et al. Complement pathway gene activation and rising circulating immune complexes characterize early disease in HIV-associated tuberculosis. Proc Natl Acad Sci U S A. 2018;115(5):E964-E73.
4. Blankley S, Graham CM, Turner J, Berry MP, Bloom CI, Xu Z, et al. The Transcriptional Signature of Active Tuberculosis Reflects Symptom Status in Extra-Pulmonary and Pulmonary Tuberculosis. PLoS One. 2016;11(10):e0162220.
