# Supplementary material for: Systematic comparison of differential expression networks in MTB mono-, HIV mono- and MTB/HIV co-infections for drug repurposing
Source: PLoS Comput Biol. 2022 Dec 19;18(12):e1010744. doi: 10.1371/journal.pcbi.1010744 (PMC9810203; doi:10.1371/journal.pcbi.1010744)
Supplement: S2 Table — (PDF) [file pcbi.1010744.s013.pdf]

S2 Table. Overlap between identified genes and existing resources

	434 genes interacting with HIV [1]	3757 genes from HIV-1-human interaction database [2]	307 genes associated with PTB [3]	933 PTB-related genes identified by the CDEN [4]
MMI-G (1007)	--	--	104 (4.81e-54)	283 (5.88e-139)
HMI-G (1266)	87 (1.46e-19)	554 (8.72e-83)	--	--
MHCI-G (1576)	125 (7.52e-35)	737 (1.11e-131)	68 (3.67e-13)	370 (4.91e-159)

p-values in parentheses are evaluated using hypergeometric tests.

References

1. Jager S, Cimermancic P, Gulbahce N, Johnson JR, McGovern KE, Clarke SC, et al. Global landscape of HIV-human protein complexes. Nature. 2011;481(7381):365-70.
2. Ako-Adjei D, Fu W, Wallin C, Katz KS, Song G, Darji D, et al. HIV-1, human interaction database: current status and new features. Nucleic Acids Res. 2015;43(Database issue):D566-70.
3. Berry MP, Graham CM, McNab FW, Xu Z, Bloch SA, Oni T, et al. An interferon-inducible neutrophil-driven blood transcriptional signature in human tuberculosis. Nature. 2010;466(7309):973-7.
4. Sun J, Shi Q, Chen X, Liu R. Decoding the similarities and specific differences between latent and active tuberculosis infections based on consistently differential expression networks. Brief Bioinform. 2020;21(6):2084-98.
