# Supplementary material for: Systematic comparison of differential expression networks in MTB mono-, HIV mono- and MTB/HIV co-infections for drug repurposing
Source: PLoS Comput Biol. 2022 Dec 19;18(12):e1010744. doi: 10.1371/journal.pcbi.1010744 (PMC9810203; doi:10.1371/journal.pcbi.1010744)
Supplement: S3 Table — (PDF) [file pcbi.1010744.s014.pdf]

S3 Table. Number of IDEN genes enriched in functionally important gene sets

Status	Gene group	Number	HIV-kegg/TB-kegg	F/B/V	E/H/I
			212/180	609/3394/5695	1575/3659/2828
HMI	DCA-G	1115	34/23	95/341/700	272/464/198
	DEA-G	209	8/1	17/34/118	67/16/36
	HMI-G	1266	40/24	108/371/774	309/474/226
MMI	DCA-G	818	22/10	77/304/588	202/389/158
	DEA-G	217	7/8	10/74/103	5/15/83
	MMI-G	1007	28/16	85/369/674	207/399/233
MHCI	DCA-G	1524	42/31	138/518/987	365/679/287
	DEA-G	67	2/2	3/21/38	2/3/25
	MHCI-G	1576	44/32	139/534/1014	367/680/308

HIV-kegg: HIV-1 pathway genes, TB-kegg: TB pathway genes, F: genes targeted by fungi, B: genes targeted by bacteria, V: genes targeted by viruses, E: essential genes, H: housekeeping genes, and I: inflammatory genes.
