# Supplementary material for: Systematic comparison of differential expression networks in MTB mono-, HIV mono- and MTB/HIV co-infections for drug repurposing
Source: PLoS Comput Biol. 2022 Dec 19;18(12):e1010744. doi: 10.1371/journal.pcbi.1010744 (PMC9810203; doi:10.1371/journal.pcbi.1010744)
Supplement: S4 Table — (PDF) [file pcbi.1010744.s015.pdf]

S4 Table. Number of subclass genes enriched in functionally important gene sets

Gene group	Number	HIV-kegg/TB-kegg	F/B/V	E/H/I
		212/180	609/3394/5695	1575/3659/2828
MMI-SG	347	7/5	24/117/188	54/96/83
HMI-SG	593	16/7	43/133/296	130/176/84
MHCI-SG	668	17/15	54/204/369	129/282/106
HMI-MMI-SG	76	4/1	8/23/49	13/33/16
HMI-MHCI -SG	324	10/7	32/101/209	98/127/68
MMI-MHCI-SG	311	7/1	27/115/215	72/131/76
Common-G	273	10/9	25/112/219	68/137/57
IDEN-G	2592	71/45	214/807/1548	564/986/490
Control-G	15549	136/129	390/2525/4084	1001/2628/2044

HIV-kegg: HIV-1 pathway genes, TB-kegg: TB pathway genes, F: genes targeted by fungi, B: genes targeted by bacteria, V: genes targeted by viruses, E: essential genes, H: housekeeping genes, and I: inflammatory genes.
