# Supplementary material for: Systematic comparison of differential expression networks in MTB mono-, HIV mono- and MTB/HIV co-infections for drug repurposing
Source: PLoS Comput Biol. 2022 Dec 19;18(12):e1010744. doi: 10.1371/journal.pcbi.1010744 (PMC9810203; doi:10.1371/journal.pcbi.1010744)
Supplement: S5 Table — (PDF) [file pcbi.1010744.s016.pdf]

S5 Table. Performance of genes and gene pairs for classification of different samples

Signature	Classification	No. of samples	Recall	Precision	F1	MCC	Accuracy	AUC
MMI-G	MMI|HC	45|61	0.933	0.977	0.955	0.923	0.962	0.983
HMI-G	HMI|HC	30|17	0.967	0.936	0.951	0.861	0.937	0.910
MHCI-G	MHCI|HC	15|61	0.867	1.00	0.929	0.916	0.974	0.993
MMI-P	MMI|HC	45|61	0.978	0.957	0.967	0.942	0.972	0.978
HMI-P	HMI|HC	30|17	0.967	0.936	0.951	0.861	0.936	0.929
MHCI-P	MHCI|HC	15|61	1.0	1.0	1.0	1.0	1.0	1.0
