# Supplementary material for: Systematic comparison of differential expression networks in MTB mono-, HIV mono- and MTB/HIV co-infections for drug repurposing
Source: PLoS Comput Biol. 2022 Dec 19;18(12):e1010744. doi: 10.1371/journal.pcbi.1010744 (PMC9810203; doi:10.1371/journal.pcbi.1010744)
Supplement: S6 Table — (PDF) [file pcbi.1010744.s017.pdf]

S6 Table. AUC values achieved by different gene signatures

PubMed ID	Number of genes	MHCI|HC	HMI|HC	MMI|HC
24587128	251	0.946	0.907	0.976
31306460	10	0.951	0.912	0.977
20725040	393	0.973	0.906	0.977
24167453	44	0.963	0.920	0.978
33816327	4	0.898	0.881	0.970
28065665	10	0.910	0.919	0.966
28649431	380	0.978	0.909	0.974
Our study	1576/1266/1007	0.993	0.910	0.983

Because our gene signature is specific to each disease state, the numbers of genes are different.
