# Supplementary material for: Systematic comparison of differential expression networks in MTB mono-, HIV mono- and MTB/HIV co-infections for drug repurposing
Source: PLoS Comput Biol. 2022 Dec 19;18(12):e1010744. doi: 10.1371/journal.pcbi.1010744 (PMC9810203; doi:10.1371/journal.pcbi.1010744)
Supplement: S7 Table — (PDF) [file pcbi.1010744.s018.pdf]

S7 Table. Biological functions of hub genes in different subnetworks

Subnetwork	Hub gene	Related function
Common-P	*SNW1*, *SF3B4*, *RNF4*, *RIOK1*, *RUNX1*	Cell cycle, endoplasmic reticulum stress, transcriptional regulation, NF-κB pathway, alternative splicing, repair of DNA damage
HMI-MMI-SP	*SNW1*, *ISG15*, *WDR5*, *IFIT1*, *DIMT1*	Interferon signaling pathway
MMI-MHCI-SP	*PSMA3*, *ISG15*, *LARP7*, *PSMA6*, *STAT1*	Proteasome activity
HMI-MHCI-SP	*ESR2*, *H4C9*, *TP53BP1*, *LMNA*, *NONO*	Repair of DNA damage
HMI-SP	*DLGAP5*, *MCM2*, *PCNA*, *TOX4*, *AURKA*	Cell cycle
MMI-SP	*TRIM25*, *EIF2AK2*, *H4C8*, *FBXO6*, *LRRK2*	Endoplasmic reticulum stress
MHCI-SP	*LARP7*, *PSMA6*, *CANX*, *RAB1A*, *ESR2*	Transcriptional regulation
