# Supplementary material for: Systematic comparison of differential expression networks in MTB mono-, HIV mono- and MTB/HIV co-infections for drug repurposing
Source: PLoS Comput Biol. 2022 Dec 19;18(12):e1010744. doi: 10.1371/journal.pcbi.1010744 (PMC9810203; doi:10.1371/journal.pcbi.1010744)
Supplement: S8 Table — (PDF) [file pcbi.1010744.s019.pdf]

S8A Table. Recall at the top K drugs of different methods for HIV drugs

Method	100	200	300	400	500	600	700	800	900	1000
Commute time	0.026	0.132	0.184	0.184	0.263	0.289	0.395	0.526	0.632	0.684
Diffusion	0.026	0.079	0.079	0.105	0.184	0.184	0.237	0.368	0.395	0.447
p-step	0.026	0.026	0.105	0.158	0.158	0.211	0.263	0.368	0.395	0.447
Inverse	0.026	0.079	0.079	0.132	0.158	0.237	0.342	0.368	0.421	0.474
Regularized	0.026	0.079	0.105	0.105	0.158	0.184	0.237	0.368	0.395	0.447
avgRank	0.053	0.053	0.105	0.211	0.237	0.289	0.289	0.342	0.421	0.474
Zhou	0.000	0.000	0.026	0.026	0.132	0.211	0.237	0.237	0.263	0.316
Guney	0.026	0.079	0.132	0.132	0.211	0.211	0.237	0.316	0.395	0.395
Distance1	0.026	0.105	0.237	0.289	0.316	0.316	0.395	0.421	0.421	0.553
Distance2	0.053	0.132	0.158	0.263	0.289	0.342	0.395	0.526	0.658	0.711
Distance3	0.000	0.079	0.263	0.289	0.316	0.316	0.500	0.553	0.579	0.684
DSD	0.079	0.237	0.237	0.368	0.474	0.632	0.658	0.684	0.684	0.737

The recall measure is the proportion of observed known drugs among all known drugs.

S8B Table. Precision at the top K drugs of different methods for HIV drugs

Method	100	200	300	400	500	600	700	800	900	1000
Commute time	0.010	0.025	0.023	0.018	0.020	0.018	0.021	0.025	0.027	0.026
Diffusion	0.010	0.015	0.010	0.010	0.014	0.012	0.013	0.018	0.017	0.017
p-step	0.010	0.005	0.013	0.015	0.012	0.013	0.014	0.018	0.017	0.017
Inverse	0.010	0.015	0.010	0.013	0.012	0.015	0.019	0.018	0.018	0.018
Regularized	0.010	0.015	0.013	0.010	0.012	0.012	0.013	0.018	0.017	0.017
avgRank	0.020	0.010	0.013	0.020	0.018	0.018	0.016	0.016	0.018	0.018
Zhou	0.000	0.000	0.003	0.003	0.010	0.013	0.013	0.011	0.011	0.012
Guney	0.010	0.015	0.017	0.013	0.016	0.013	0.013	0.015	0.017	0.015
Distance1	0.010	0.020	0.030	0.028	0.024	0.020	0.021	0.020	0.018	0.021
Distance2	0.020	0.025	0.020	0.025	0.022	0.022	0.021	0.025	0.028	0.027
Distance3	0.000	0.015	0.033	0.028	0.024	0.020	0.027	0.026	0.024	0.026
DSD	0.030	0.045	0.030	0.035	0.036	0.040	0.036	0.033	0.029	0.028

The precision measure is the proportion of observed known drugs among the top K drugs.
