# Supplementary material for: Systematic comparison of differential expression networks in MTB mono-, HIV mono- and MTB/HIV co-infections for drug repurposing
Source: PLoS Comput Biol. 2022 Dec 19;18(12):e1010744. doi: 10.1371/journal.pcbi.1010744 (PMC9810203; doi:10.1371/journal.pcbi.1010744)
Supplement: S9 Table — (PDF) [file pcbi.1010744.s020.pdf]

S9A Table. Recall at the top K drugs of different methods for TB drugs

Method	100	200	300	400	500	600	700	800	900	1000
Commute time	0.000	0.000	0.133	0.133	0.133	0.200	0.267	0.267	0.267	0.267
Diffusion	0.000	0.000	0.000	0.067	0.067	0.200	0.333	0.333	0.467	0.467
p-step	0.000	0.000	0.000	0.067	0.200	0.200	0.333	0.333	0.467	0.467
Inverse	0.000	0.000	0.000	0.133	0.133	0.200	0.200	0.267	0.333	0.400
Regularized	0.000	0.000	0.000	0.067	0.133	0.200	0.267	0.333	0.467	0.467
avgRank	0.000	0.000	0.000	0.067	0.067	0.133	0.133	0.200	0.467	0.467
Zhou	0.000	0.000	0.000	0.000	0.000	0.000	0.000	0.067	0.133	0.133
Guney	0.067	0.133	0.133	0.133	0.133	0.200	0.200	0.200	0.200	0.200
Distance1	0.067	0.133	0.200	0.200	0.200	0.200	0.200	0.333	0.333	0.333
Distance2	0.000	0.067	0.133	0.267	0.333	0.400	0.467	0.733	0.933	0.933
Distance3	0.067	0.067	0.133	0.267	0.267	0.267	0.267	0.333	0.333	0.467
DSD	0.067	0.467	0.600	0.733	0.800	0.867	0.933	0.933	0.933	0.933

The recall measure is the proportion of observed known drugs among all known drugs.

S9B Table. Precision at the top K drugs of different methods for TB drugs

Method	100	200	300	400	500	600	700	800	900	1000
Commute time	0.000	0.000	0.007	0.005	0.004	0.005	0.006	0.005	0.004	0.004
Diffusion	0.000	0.000	0.000	0.003	0.002	0.005	0.007	0.006	0.008	0.007
p-step	0.000	0.000	0.000	0.003	0.006	0.005	0.007	0.006	0.008	0.007
Inverse	0.000	0.000	0.000	0.005	0.004	0.005	0.004	0.005	0.006	0.006
Regularized	0.000	0.000	0.000	0.003	0.004	0.005	0.006	0.006	0.008	0.007
avgRank	0.000	0.000	0.000	0.003	0.002	0.003	0.003	0.004	0.008	0.007
Zhou	0.000	0.000	0.000	0.000	0.000	0.000	0.000	0.001	0.002	0.002
Guney	0.010	0.010	0.007	0.005	0.004	0.005	0.004	0.004	0.003	0.003
Distance1	0.010	0.010	0.010	0.007	0.006	0.005	0.004	0.006	0.006	0.005
Distance2	0.000	0.005	0.007	0.010	0.010	0.010	0.010	0.014	0.016	0.014
Distance3	0.010	0.005	0.007	0.010	0.008	0.007	0.006	0.006	0.006	0.007
DSD	0.010	0.035	0.030	0.028	0.024	0.022	0.020	0.018	0.016	0.014

The precision measure is the proportion of observed known drugs among the top K drugs.
