# Supplementary material for: Systematic comparison of differential expression networks in MTB mono-, HIV mono- and MTB/HIV co-infections for drug repurposing
Source: PLoS Comput Biol. 2022 Dec 19;18(12):e1010744. doi: 10.1371/journal.pcbi.1010744 (PMC9810203; doi:10.1371/journal.pcbi.1010744)
Supplement: S10 Table — (PDF) [file pcbi.1010744.s021.pdf]

**S10 Table. Repurposed anti-MMI drug candidates**

| DrugBank ID | Drug | Distance | Z-score | GSEA score | Pharmacological category | Known indication | PubMed ID |
|---|---|---|---|---|---|---|---|
| DB06401 | Bazedoxifene | 3.09 | -2.40 | NA | Selective estrogen receptor modulator | Postmenopausal osteoporosis | 32269154 |
| DB00783 | Estradiol | 3.07 | -2.38 | -20.65 | Estrogenic steroid | Hypoestrogenism | 35425995 |
| DB00619 | Imatinib | 3.18 | -2.22 | NA | Antineoplastic agent | Gastrointestinal stromal tumor | 19251803 |
| DB00440 | Trimethoprim | 4.42 | -2.07 | NA | Antifolate antibiotic | Urinary tract infection, respiratory tract infection | 25907064<br>25246405<br>22825115 |
| DB00539 | Toremifene | 3.46 | -1.19 | -11.21 | Selective estrogen receptor modulator | Breast cancer | 11937596 |
| DB00207 | Azithromycin | 4.76 | -1.18 | NA | Macrolide antibiotic | Bacterial infection | 7490437 |
| DB01165 | Ofloxacin | 4.04 | -1.09 | NA | Fluoroquinolone antibacterial agent | Bacterial infection | 26195507<br>14555582 |
| DB00537 | Ciprofloxacin | 4.00 | -1.06 | NA | Fluoroquinolone antibacterial agent | Bacterial infection | 24440548<br>11217874 |
| DB00218 | Moxifloxacin | 4.04 | -1.05 | -0.60 | Fluoroquinolone antibacterial agent | Bacterial infection | 30385322<br>35146045<br>2918650 |
| DB01208 | Sparfloxacin | 4.04 | -1.05 | NA | Fluoroquinolone antibacterial agent | Bacterial respiratory infection | 21834759<br>11401058 |
