# Supplementary material for: Systematic comparison of differential expression networks in MTB mono-, HIV mono- and MTB/HIV co-infections for drug repurposing
Source: PLoS Comput Biol. 2022 Dec 19;18(12):e1010744. doi: 10.1371/journal.pcbi.1010744 (PMC9810203; doi:10.1371/journal.pcbi.1010744)
Supplement: S11 Table — (PDF) [file pcbi.1010744.s022.pdf]

S11 Table. Repurposed anti-HMI drug candidates

DrugBank ID	Drug	Distance	Z-score	GSEA score	Pharmacological category	Known indication	PubMed ID
DB01073	Fludarabine	3.95	-2.41	-27.92	Antimetabolite	Chronic lymphocytic leukemia	20568281 18003888
DB01029	Irbesartan	3.32	-2.37	-0.46	Angiotensin receptor blocker	Hypertension, diabetic nephropathy	20516556
DB00631	Clofarabine	3.93	-2.28	-92.83	Antimetabolite	Relapsed or refractory acute lymphoblastic leukemia	27009333 25103850 28220857
DB00242	Cladribine	3.80	-2.22	-62.86	Antimetabolite	Lymphoproliferative disease	31644243
DB00762	Irinotecan	3.87	-1.57	-57.3	Antineoplastic agent	Colorectal cancer	18596388
DB01030	Topotecan	3.87	-1.54	-97.43	Antineoplastic agent	Ovarian cancer	11363913 11362319 9145855
DB00441	Gemcitabine	4.03	-1.35	-85.26	Nucleoside metabolic inhibitor	Pancreatic cancer	23994876 22271861 21264291
DB00481	Raloxifene	3.22	-1.21	NA	Selective estrogen receptor modulator	Osteoporosis	18413693 30061382
DB00619	Imatinib	3.27	-1.20	-6.53	Antineoplastic agent	Gastrointestinal stromal tumor	19251803
DB00783	Estradiol	3.20	-1.15	-25.34	Estrogenic steroid	Hypoestrogenism	22827217
