# Supplementary material for: Systematic comparison of differential expression networks in MTB mono-, HIV mono- and MTB/HIV co-infections for drug repurposing
Source: PLoS Comput Biol. 2022 Dec 19;18(12):e1010744. doi: 10.1371/journal.pcbi.1010744 (PMC9810203; doi:10.1371/journal.pcbi.1010744)
Supplement: S12 Table — (PDF) [file pcbi.1010744.s023.pdf]

S12 Table. Repurposed anti-MHCI drug candidates

DrugBank ID	Drug	Distance	Z-score	GSEA score	Pharmacological category	Known indication	PubMed ID
DB00570	Vinblastine	3.04	-2.90	-27.6	Antineoplastic agent	Breast cancer, testicular cancer	33725144<br>20167961
DB01029	Irbesartan	3.20	-2.81	NA	Angiotensin receptor blocker	Hypertension, diabetic nephropathy	20516556
DB00783	Estradiol	3.00	-2.57	-40.06	Estrogenic steroid	Hypoestrogenism	22827217
DB00619	Imatinib	3.14	-2.38	NA	Antineoplastic agent	Gastrointestinal stromal tumor	19251803
DB00440	Trimethoprim	4.43	-1.96	NA	Antifolate antibiotic	Urinary tract infection, respiratory tract infection	25907064<br>25246405<br>22825115
DB04216	Quercetin	4.26	-1.70	NA	Antioxidant	Inflammation	34709675
DB01030	Topotecan	3.86	-1.61	NA	Antineoplastic agent	Ovarian cancer	11363913<br>11362319<br>9145855
DB00762	Irinotecan	3.86	-1.57	NA	Antineoplastic agent	Colorectal cancer	18596388
DB00361	Vinorelbine	3.74	-1.35	-3.1	Antineoplastic agent	Non-small cell lung carcinoma	10348092<br>11342309
DB00537	Ciprofloxacin	3.97	-1.25	NA	Antibacterial agent	Bacterial infection	24440548<br>11217874
DB00539	Toremifene	3.44	-1.13	NA	Selective estrogen receptor modulator	Breast cancer	11937596
